# Synthesis and characterization of *S,N*-heterotetracenes

**DOI:** 10.3762/bjoc.16.214

**Published:** 2020-10-26

**Authors:** Astrid Vogt, Florian Henne, Christoph Wetzel, Elena Mena-Osteritz, Peter Bäuerle

**Affiliations:** 1 Institute of Organic Chemistry II and Advanced Materials, University of Ulm, Albert-Einstein-Allee 11, 89081 Ulm, Germany; 2 Carl Zeiss SMT GmbH, Rudolf-Eber-Straße 2, 73447 Oberkochen, Germany; 3 Merck KGaA, Frankfurter Str. 250, 64293 Darmstadt, Germany

**Keywords:** Buchwald–Hartwig amination, Cadogan reaction, optoelectronic properties, *S,N*-heteroacene, structure–property relationship

## Abstract

The synthesis and optoelectronic properties of novel *S,N*-heterotetracenes consisting of fused heterocyclic thiophene and pyrrole rings are presented. Tetracyclic and benzannulated derivatives with a varying number and sequence of sulfur and nitrogen heteroatoms were synthesized in multistep synthetic routes. A Buchwald–Hartwig amination of brominated precursors, thermolysis of azide precursors, and a Cadogan reaction of nitro-substituted precursors were successfully applied to eventually build-up pyrrole rings to stable and soluble fused systems. The various obtained heteroatom sequences ‘SSNS*’* (SN4), ‘SNNS*’* (SN4’’), and ‘NSSN*’* (SN4’) allowed for evaluation of structure–property relationships relative to the sulfur analogue tetrathienoacene (‘SSSS*’*). In line with the results for the whole series of *S,N*-heteroacenes, we find that replacement of sulfur by nitrogen atoms in the tetra- and hexacyclic systems leads to a red-shift in absorption, a decrease in oxidation potential and energy gap. On the other hand, the replacement of a thiophene ring by benzene leads to the opposite effects.

## Introduction

Thienoacenes and related *S,N*-heteroacenes have been developed to important π-conjugated systems mainly for application as p-type semiconductor in organic electronic devices with excellent results [1–3]. The series of thienoacenes consisting of only fused thiophene rings has been synthesized up to an octamer, whereby the longer members in the series quickly lose solubility [4–6]. The implementation of pyrrole rings into the fused ladder-type structure leads to the class of *S,N*-heteroacenes, whereby the incorporation of the electron-rich pyrrole rings not only modifies the electronic properties compared to thienoacenes, but also allows for the attachment of side chains at the pyrrole nitrogen as a useful synthetic tool to enhance solubility [7]. The smaller members of the series of *S,N*-heteroacenes, dimeric thieno[3,2-*b*]pyrrole [8] and trimeric dithieno[3,2-*b*:2’,3’-*d*]pyrrole (DTP) [9], are long known but still frequently used as building block for organic electronic materials [10]. By application of Pd-catalyzed Buchwald–Hartwig amination/cyclization reactions of brominated thiophene-based precursors and amines [11–12], the series of *S,N*-heteroacenes has been systematically extended from pentamer SN5 [13–14] up to a stable and still soluble *S,N*-heterotridecacene SN13 [7,15–17]. Correlation of, e.g., absorption or redox potentials vs conjugated chain length led to interesting structure–property relationships showing the influence of the number and sequence of pyrrole rings. The highly planar π-conjugated systems revealed interesting structural features such as nearly complete bond length equalization and S–S and S–π dipolar interactions in the solid state. Due to tunable optoelectronic properties, a variety of functionalized derivatives, mainly trimeric DTP (SN3) [18], pentameric SN5 [19–23], and hexameric SN6 [24–28], have been successfully implemented as active semiconducting components in organic solar cells [18–21,24–26], perovskite solar cells [22,27], organic field-effect transistors [28], or sensors [23]. The interesting structure–property relationships of the *S,N*-heteroacene series inspired as well theoretical work. In this respect, De Simone et al. computed electronic spectra of the neutral, charged radical cationic, and dicationic species [29], whereas Sarkar et al. undertook quantum chemical investigations on the photovoltaic performance revealing an odd-even relation of the charge separation [30]. Furthermore, they calculated the single molecule transport behavior of various *S,N*-heteroacenes with different conjugation length between nanocontacts [31].

**Figure 1:**
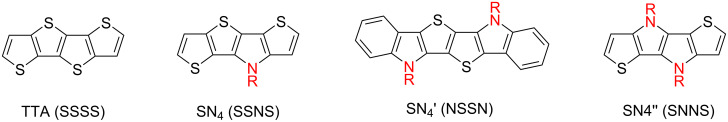
Heteroacenes: tetrathienoacene (TTA), *S*,*N*-heteroacenes SN4, SN4', and SN4''.

The obvious missing link in the series of *S,N*-heteroacenes is represented by heterotetrameric SN4, which is very rarely described in the literature. SN4 is derived from well-known parent tetrathienoacene (TTA, ‘SSSS*’*, Figure 1) by replacing one “S” by “N” or in other words one fused thiophene unit by a pyrrole ring. For TTA various synthetic methods have been developed and it has been broadly implemented as building block into materials for organic electronic devices [32–33]. We have disclosed the first SN4 derivative with the heteroatom sequence ‘SSNS*’* in the context of complementing the series, but did not yet describe synthetic details [7]. Xue et al. published a donor–acceptor photosensitizer for dye-sensitized solar cells, in which the *N*-phenylated SN4 unit serves as bridge between donor and acceptor moieties [34]. Further replacement of sulfur by nitrogen can lead to different isomeric SN4 structures with possible sequences ‘NSSN’, which we denominate as SN4', and ‘SNNS’ as SN4'' (Figure 1). In this respect, the only known structures were prepared by Gryko et al., who synthesized heterohexacenes with *N*-phenylated SN4'' cores [35]. The same group furthermore prepared heterotetracenes comprising four pyrrole rings (‘NNNN’) as electron-rich, ladder-type fluorophores by a two-step synthesis [36]. The possible asymmetric SN4 isomer ‘SNSN’ and heterotetracenes comprising three pyrrole rings are unknown so far.

In continuation of our work on heteroacenes, we now report in detail the syntheses and characterization of fused *S,N*-heterotetracenes, SN4 (‘SSNS*’*), benzannulated SN4' (‘NSSN*’*), and SN4'' (‘SNNS*’*) by application of various cyclization methods to build up pyrrole rings in the tetraheterocyclic systems. Among them, a Buchwald–Hartwig amination of brominated precursors, thermolysis of azide precursors, and a Cadogan cyclization of nitro-substituted precursors were applied to prepare various unknown derivatives. These represent novel core molecules, which by further derivatisation, for example with terminal acceptor groups, would lead to interesting π-conjugated materials for application in organic electronics.

## Results and Discussion

**Scheme 1:**
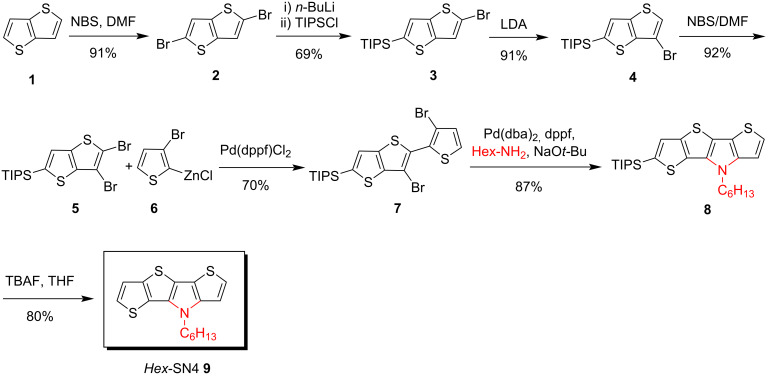
Synthesis of fused *S,N*-heterotetracene SN4 **9** starting from thieno[3,2-*b*]thiophene (**1**).

**Synthesis of *N*-alkylated *S,N*-heterotetracene 9 by Pd-catalyzed amination***.* Asymmetric *S,N*-heterotetracene *Hex*-SN4 **9** comprises the sequence of heteroatoms ‘SSNS’ in the tetracyclic conjugated π-system [37]. In a multistep synthesis, 2,6-dibromothienothiophene **2** [39], which was obtained from thieno[3,2-*b*]thiophene (**1**) [38], was triisopropylsilyl (TIPS)-protected by lithium-halogen exchange with *n*-BuLi and triisopropylsilyl chloride to give thienothiophene **3** in 69% yield. A halogen dance reaction of **3**, induced by lithium diisopropylamide (LDA) at −78 °C, gave the corresponding β-brominated thienothiophene **4** in 91% yield [6]. Compound **4** was selectively brominated in α-position by using NBS in DMF at room temperature to give dibrominated thienothiophene **5** in 92% yield which was coupled in a Negishi-type cross-coupling reaction with zincated 2,3-dibromothiophene **6** and Pd(dppf)Cl_2_ as catalyst to give the corresponding dibromide **7** in a yield of 70%. Organozinc species **6** was obtained from 2,3-dibromothiophene by lithiation with *n*-BuLi and reaction with zink dichloride. Annulation to TIPS-protected SN4-Hex **8** was achieved in 87% yield by a tandem Buchwald–Hartwig amination of dibromide **7** with *n*-hexylamine in the presence of sodium *tert*-butoxide as base and Pd(dba)_2_/dppf as the catalytic system. The TIPS group was removed from *Hex*-SN4 **8** upon treatment with tetra-*n*-butylammonium fluoride (TBAF) and the parent system *Hex*-SN4 **9** was obtained in 80% yield (Scheme 1).

**Scheme 2:**
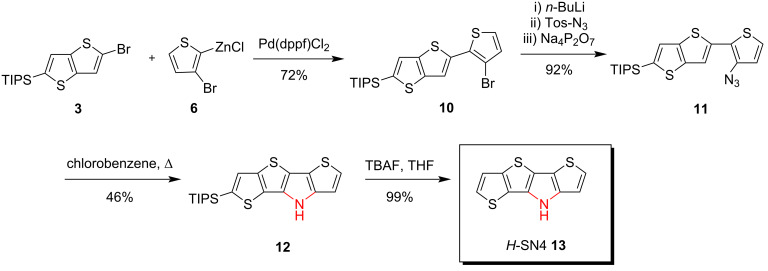
Synthesis of parent *H*-SN4 **13** via the azide route.

**Synthesis of *S,N*-heterotetracene *H*-SN4 13 by thermolysis of azide precursors***.* Smaller parent heterotriacene dithienopyrrole (*H*-DTP) was first synthesized by Zanirato et al. by thermolysis of 3-azido-2,2’-bithiophene as the key step [9]. We therefore tried to build up tetracyclic *H*-SN4 **13** via the azide route. The synthesis started from the above mentioned brominated thieno[3,2-*b*]thiophene **3**, which was coupled with zincated 2,3-dibromothiophene **6** in a Negishi-type cross-coupling reaction with Pd(dppf)Cl_2_ as catalyst to give thiophene-substituted thienothiophene **10** in 72% yield. In the next step, an azide group was introduced to **10** by lithiation with *n*-BuLi followed by the reaction with tosyl azide giving azide **11** in an excellent yield of 92%. Thermally induced ring-closure under release of nitrogen and formation of a nitrene intermediate was achieved by refluxing azide **11** in chlorobenzene for 30 min to yield TIPS-protected *H*-SN4 **12** in 46% yield. Finally, the TIPS group of *S,N*-heterotetracene **12** was smoothly removed using TBAF in THF solution to yield the basic structure *H*-SN4 **13** in almost quantitative yield (Scheme 2).

**Scheme 3:**
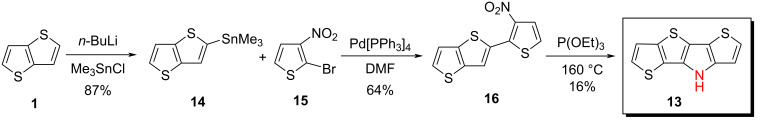
Synthesis of tetracyclic *H*-SN4 **13** via the Cadogan route.

**Synthesis of *S,N*-heterotetracene *H*-SN4 13 and related derivatives by Cadogan reaction of nitro-substituted precursors***.* Another possibility for the preparation of fused pyrrole rings is established by the Cadogan reaction [40]. Well-known examples are the reductive cyclization of 2-nitro-1,1’-biphenyls with triethyl phosphite or phosphanes as reducing agent to provide carbazoles covering a wide substrate and functional group spectrum [41]. Moreover, penta- and heptafused heteroacenes were prepared by the Cadogan reaction by annulation of nitrophenyl or nitrobenzothienyl precursors [42–45]. In this respect, we recently reported a Cadogan cyclization of 3-nitro-2,2’-bithiophene with triethyl phosphite under microwave irradiation and surprisingly obtained targeted heterotriacene *H*-DTP (vide supra*)* with only 11% yield [45]. This result prompted us to have a closer look on the applicability of the Cadogan reaction/cyclization in order to provide *S,N*-heterotetracenes and related derivatives from nitro-substituted thiophene-based precursors. First of all, the reductive cyclization of nitrothienyl-substituted thienothiophene **16** to *H*-SN4 **13** was investigated. For this purpose, 2-stannylthienothiophene **14**, which was obtained from thienothiophene **1**, was converted in a Pd-catalyzed Stille-type cross-coupling with 2-bromo-3-nitrothiophene (**15**) to precursor 2-(3-nitrothien-2-yl)thieno[3,2-*b*]thiophene (**16**) in 64% yield. Cadogan cyclization of nitro derivative **16** with triethyl phosphite at reflux gave 4*H*-thieno[3,2-*b*]thieno[2’, 3’:4,5]thieno[2,3-*d*]pyrrole (*H*-SN4, **13**) in a similar low yield (16%) as before *H*-DTP (vide supra) (Scheme 3). The change of the reducing agent to triphenyl phosphite did not lead to a reaction at all.

**Scheme 4:**
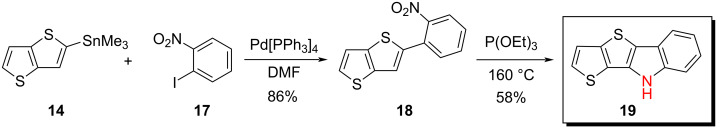
Synthesis of tetracyclic indole derivative **19** via the Cadogan route.

We turned then to related thienothiophene **18**, which bears an *o*-nitrophenyl substituent instead of a 3-nitrothienyl residue in **16**. 2-(2-Nitrophenyl)thieno[3,2-*b*]thiophene (**18**) was similarly prepared by Pd-catalyzed Stille-type reaction of monostannylated thienothiophene **14** and *o*-iodonitrobenzene (**17**) in 86% yield. Successive Cadogan cyclization of **18** with triethyl phosphite under reflux gave tetracyclic 9*H*-thieno[2’,3’:4,5]thieno[3,2-*b*]indole (**19**) in an increased yield of 58% (Scheme 4).

Comparison of the reactions indicated that *o*-nitrophenyl substituents as in **18** give higher cyclization yields than the corresponding 3-nitrothienyls as in **16**. We rationalize this finding by a higher reactivity of the intermediate thienyl nitrene, which is formed during the cyclization reaction of **16** compared to phenyl nitrene [42]. Besides *H*-SN4 **13**, which is formed by insertion of the nitrene N-atom into the C_3_–H σ-bond of the thienothiophene in **16** and simultaneous migration of the H-atom to the nitrogen, mostly undefined black oligomeric or polymeric products were obtained.

**Scheme 5:**
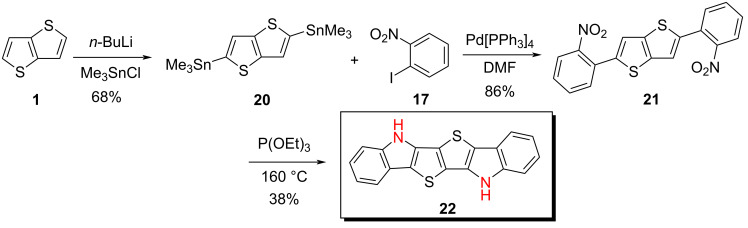
Synthesis of hexacyclic heteroacene SN4' **22** via the Cadogan route.

We further tested a twofold nitrophenyl-substituted thienothiophene in a Cadogan cyclization. Thus, distannylated thienothiophene **20** was obtained in 68% yield from thienothiophene **1** with two equivalents each of *n*-BuLi at −78 °C and trimethylstannyl chloride in THF. The stannyl reacted with *o*-iodonitrobenzene (**17**) and Pd[PPh_3_]_4_ as catalyst in DMF to give 2,5-bis(2-nitrophenyl)thieno[3,2-*b*]thiophene (**21**) in 86% yield. Subsequent Cadogan cyclization with triethyl phosphite under reflux gave hexacyclic *S,N*-heteroacene **22** in 38% yield which reflects a benzannulated SN4' system with the heteroatom sequence ‘NSSN’ (Scheme 5). The use of triphenyl phosphite as reducing agent gave a decreased yield of 24%. The *N*-alkylated derivative of hexacyclic SN4' **22** was prepared by Wong et al., who applied the Cadogan reaction to cyclize a dibrominated precursor similar to **21**. Without isolation, the SN4’-intermediate was directly alkylated with 2-ethylhexyl bromide and the corresponding dialkylated SN4' system was subsequently obtained by removal of the bromines by lithium–halogen exchange and acidic work-up [28].

**Scheme 6:**
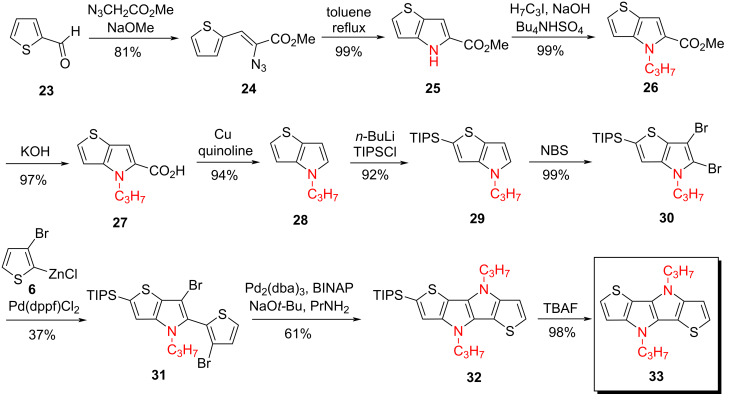
Synthesis of heterotetracene SN4'' **33** via the azide and Buchwald–Hartwig amination route.

**Synthesis of *S,N*-heterotetracenes SN4''***.* In contrast to SN4’, *S,N*-heterotetracene system SN4'' comprising the heteroatom sequence ‘SNNS’ is built-up by annulation of two ‘outer’ thiophene and two ‘inner’ pyrrole rings resulting in the symmetric sequence of heteroatoms in the tetracyclic conjugated π-system. The multistep synthesis of *Pr*-SN4'' **33** started from 2-thienylcarbaldehyde (**23**), which was put to reaction with methyl 2-azidoactetate [46] and sodium methanolate in a Knoevenagel condensation to give azide **24** in 81% yield. A solution of the azide **24** was added to boiling toluene and cyclization to thienopyrrole **25** occurred via a nitrene intermediate in nearly quantitative yield [47]. Single crystals of intermediate thienopyrrole **25** were obtained by slow evaporation of a chloroform solution and an X-ray structure analysis was performed. Structural details and packing motifs of the molecule are described in Supporting Information File 1. Alkylation to *N*-propyl-substituted thienopyrrole **26**, saponification to carbonic acid **27**, and subsequent Cu-mediated decarboxylation in quinoline resulted in thienopyrrole **28** in more than 80% yield over three steps. Lithiation of **28** with *n*-BuLi and reaction with TIPS chloride selectively occurred at the thiophene α-position to give TIPS-protected thienopyrrole **29** in 92% yield which was further brominated with *N*-bromosuccinimide (NBS) to dibrominated thienopyrrole **30** in 99% yield. Pd-catalyzed Neghishi-type coupling of **30** with 3-bromo-2-thienylzinc chloride (**6**) resulted in thienyl-substituted thienopyrrole **31** in moderate 37% yield. Cyclization to form the second pyrrole ring was achieved by selective Buchwald–Hartwig amination of dibromide **31** with *n*-propylamine to give TIPS-protected SN4'' **32** in 61% yield. Final removal of the TIPS-protecting group by TBAF gave the novel SN4''-system **33** in 98% yield (Scheme 6).

All prepared *S,N*-heterotetracenes and -hexacene were clearly identified, fully characterized, and their structures confirmed by ^1^H and ^13^C NMR spectroscopy and high-resolution mass spectrometry (HRMS) via matrix-assisted laser desorption/ionization (MALDI) (Supporting Information File 1, Figures S1–S5). In the ^1^H NMR spectra, the *S,N*-heterotetracenes typically showed two doublets with ^3^*J*-coupling constants of 5.2–5.3 Hz for the α- and β-protons of the terminal thiophene units in the fused skeleton. Thereby the α-protons typically occur slightly downfield-shifted relative to the ß-protons [20]. If one compares the shifts of these protons of the basic systems TTA (H_α_ 7.52, H_ß_ 7.41 ppm) with those of SN4 **13** (H_α_ 7.39, H_ß_ 7.33 ppm) and SN4'' **33** (H_α_ 7.06, H_ß_ 7.02 ppm), both protons gradually shift up-field the more electron-rich pyrrole rings are present in the system. In contrast, for benzofused heteroacene **19** these protons are shifted down-field (H_α_ 7.67, H_ß_ 7.49 ppm) compared to SN4 **13**. MALDI–HRMS of all synthesized *S,N*-heteroacenes showed only a single peak for the molecular ions accounting for their purity.

**Figure 2:**
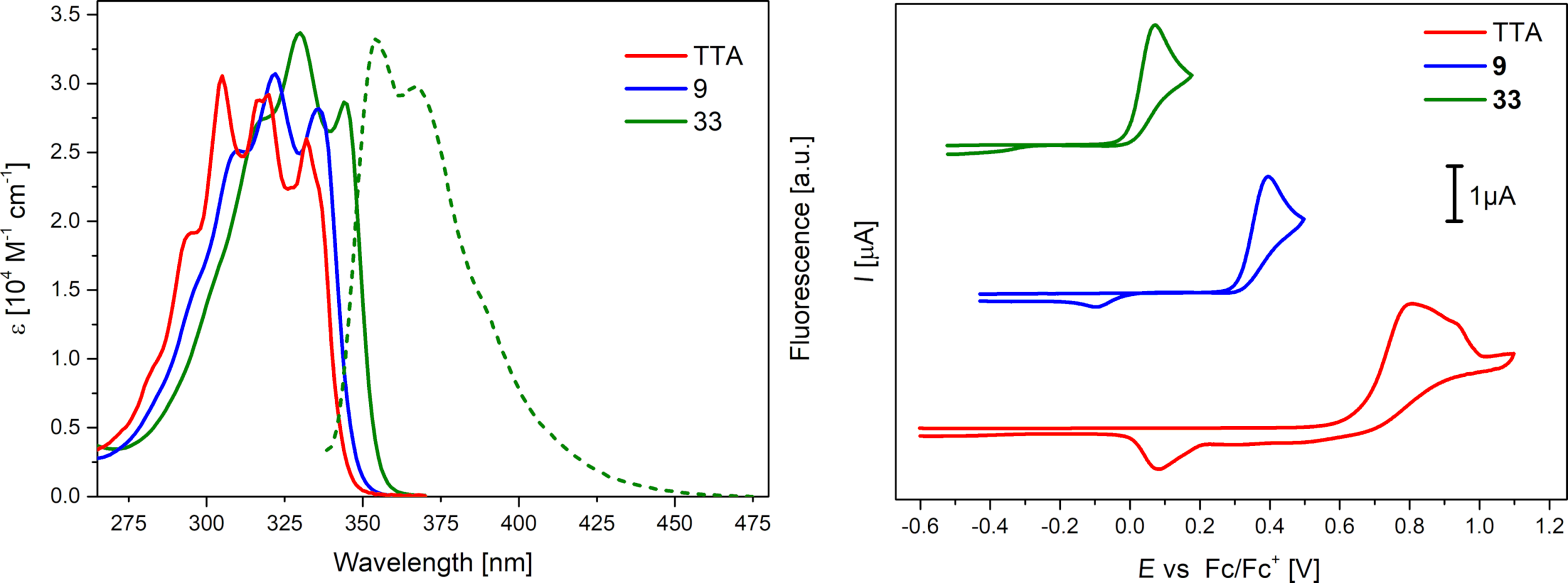
UV–vis absorption spectra of TTA, *Hex*-SN4 **9**, *Pr*-SN4'' **33** and fluorescence spectrum of **33** in THF at rt (left) and corresponding cyclic voltammograms in dichloromethane/tetrabutylammonium hexafluorophosphate (0.1 M), 100 mV/s (right).

**Optical and redox properties of *S,N*-heteroacenes 9, 13, 19, 22, and 33 in comparison to tetrathienoacene (TTA).** Optical spectroscopy of the various SN4-heteroacenes was performed in THF solutions. UV–vis absorption spectra of TTA, SN4 **9**, and SN4'' **33** are shown in Figure 2, left, those of heteroacenes **13**, **19**, and **22** in Figure S6 (Supporting Information File 1) and data is listed in Table 1. In general, the heteroacenes exhibited one vibronically well-resolved strong absorption band in the UV-regime of 250–360 nm which we address to the π–π* transition corresponding to the HOMO–LUMO energy gap. The absorption maximum is gradually red-shifted from 318 nm for TTA and with increasing number of pyrrole rings to 330 nm for SN4'' **33** including molar extinction coefficients in the range from 30 500 M^−1^ cm^−1^ for TTA to 33 700 M^−1^ cm^−1^ for SN4'' derivative **33**. In comparison to *H*-SN4 **13**, the influence of the hexyl side chain in *Hex*-SN4 **9** on the optical properties is as expected marginal and the absorption maximum is only slightly red-shifted. The effect of benzannulation in **19** compared to *H*-SN4 **13** as well results only in small differences, which, however, become larger in comparison to hexacyclic heteroacene **22** showing the largest bathochromic shift in the series (Figure S6, Supporting Information File 1). The absorption maximum (378 nm) and the shape of the band are comparable to those of SN6 (379 nm) [17]. The pronounced vibronic fine splitting of the main absorption bands is typical for the planar and stiff-fused backbones of the heteroacenes [20]. The optical gap, which is determined from the absorption onset, gradually decreases with increasing number of pyrrole rings in the heteroacenes from 3.62 eV for TTA to 3.50 eV for **33** (Figure 2, left).

The SN4-heteroacenes fluoresce, if at all, only very weakly and emission maxima lay in between 356 nm for TTA and 388 nm for **22** with the most extended conjugated π-system (Table 1). Fluorescence quantum yields were far below 1% except for hexacene **22**, which showed a very moderate value of 1.9%. This result is in line with the series of *S,N*-heteroacenes where noticeable fluorescence started with heptacene SN7 (ϕ_em_ = 19%) reaching 93% for the longest tridecamer SN13 [17,37]. The Stokes shifts, which were determined from the 0,0-transitions, lay in between 682 cm^−1^ for hexacene **22** and 1433 cm^−1^ for SN4'-derivative **9**. These relatively small values indicate small geometric changes and structural deformation between the ground and excited states which is typical for such planar and conformationally restricted systems [48].

**Table 1:** Thermal, optical, and electrochemical data of *S*,*N*-heterotetracenes **9**, **13**, and **33** in comparison to tetrathienoacene (TTA) and benzofused *S*,*N*-heteroacenes **19** and **22**.

Heteroacene	Mp [°C]	λ_abs_ [nm]^a^	ε [M^−1^ cm^−1^]	*E*_g_^opt^ [eV]^b^	λ_em_ [nm]^a^	ϕ_em_ [%]^c^	Stokes shift [cm^−1^]	*E*_p_^Ox1^ [V]	HOMO [eV]^d^	LUMO [eV]^e^

TTA	222.0	332, 318, 305	30 500	3.62	356	<<1	965^f^	0.81	−5.76	−2.14

*H*-SN4 (**13**)	186.3	333, 320, 305	29 300	3.57	n.d.^g^	n.d.^g^	n.d.^g^	0.37	−5.37	−1.80
*Hex*-SN4 (**9**)	66.3	336, 322, 310	30 700	3.55	364	<<1	1433^f^	0.39	−5.39	−1.84
*Pr*-SN4’’ (**33**)	141.0	344, 330, 317	33 700	3.50	355	<<1	861	0.07	−5.10	−1.60

*H*-SN3B (**19**)	225–226	339, 325, 313	29 500	3.55	n.d.^g^	n.d.^g^	n.d.^g^	0.56	−5.56	−2.01
*H*-BSN4’B (**22**)	452	378, 358, 336	52 500	3.20	388	1.9	682	0.33	−5.27	−2.07

^a^Absorption and emission measured in THF. ^b^Calculated by *E*_g_ = 1240/λ_onset_. ^c^Quantum yields referred to anthracene in ethanol (ϕ_em_ = 0.27). ^d^Calculated from the onset value of the oxidation wave; Fc/Fc^+^ was set to −5.1 eV vs vacuum. ^e^Calculated with *E*_HOMO_ and *E*_g_^opt^ sol. ^f^Value calculated through Gaussian deconvolution of the unstructured emission spectrum. ^g^n.d. = not detectable.

In order to get information about the redox properties and energetics of the frontier orbitals, the *S,N-*heteroacenes were studied by cyclic voltammetry and all potentials were referenced against the ferrocene/ferricenium couple (Table 1). In the series TTA, SN4 **9**, and SN4'' **33** irreversible oxidation waves were obtained due to follow-up reactions of the formed radical cations and peak potentials gradually decrease from 0.81 V for TTA to 0.07 V for SN4'' **33** (Figure 2, right). As expected, the oxidation potential is substantially influenced by the increasing number of electron-rich pyrrole rings and drops continuously. As expected, if an electron-rich fused thiophene ring in *H*-SN4 **13** is replaced by a phenyl ring in **19**, the oxidation potential is increased by 190 mV. The value for hexacyclic **22** (*E*_p_^ox1^ = 0.33 V) is in the same regime than for *H*-SN4 **13**, but substantially higher than this for SN6 (*E*_p_^ox1^ = 0.06 V) [7,37] clearly showing the effect of the two-fold benzannulation.

**Figure 3:**
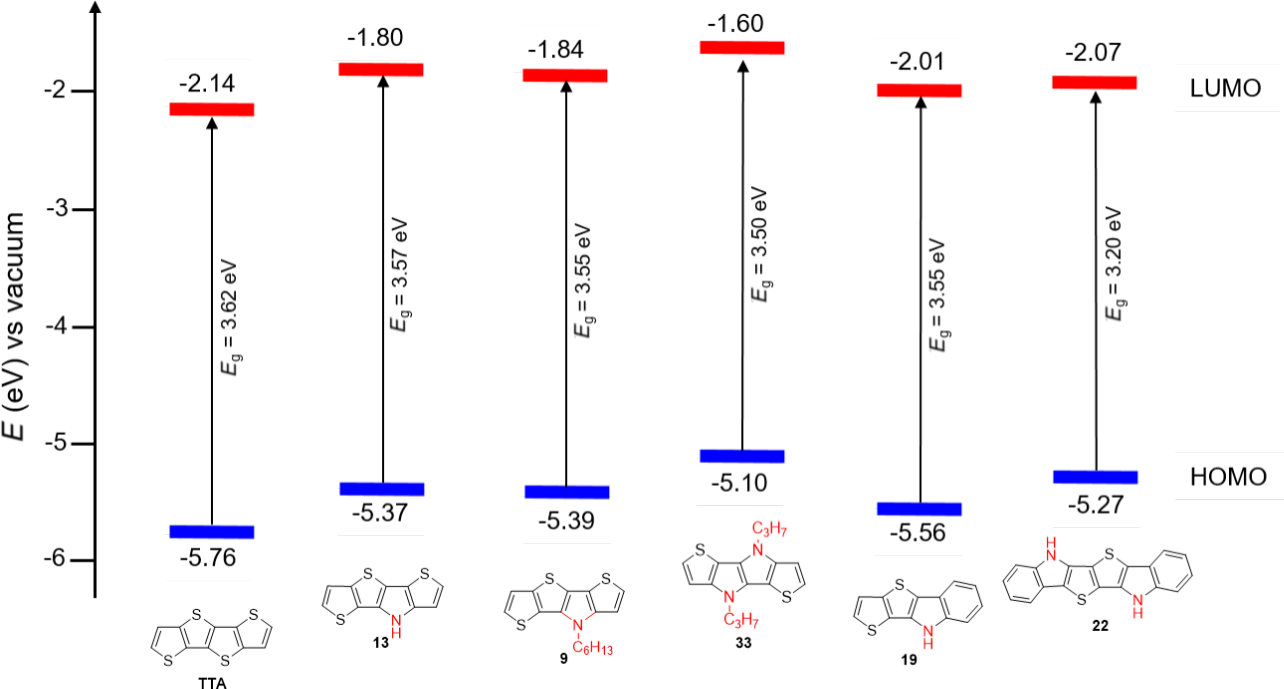
Energy diagram of the frontier molecular orbitals of heterotetracenes TTA, **9**, **13**, **19**, **22**, and **33,** and heterohexacene **22**.

From the optoelectronic data a schematic energy level diagram of the frontier orbitals and the optical transitions in the series can be derived (Figure 3). The HOMO energy levels were determined from the onset of the oxidation wave and are continuously destabilized in the series TTA, SN4 **13** and **9**, and SN4'' **33**. Due to the absence of reduction waves in the cyclic voltammograms, the LUMO energy levels were calculated from the optical energy gaps *E*_g_^opt^ and the HOMO energy levels. As well the LUMOs are destabilized with increasing number of pyrrole rings.

## Conclusion

In summary, we have presented the synthesis and characterization of novel *S,N*-heterotetracenes and -hexacene with varying the number and sequence of the sulfur and nitrogen heteroatoms which complement the general series of *S,N*-heteroacenes. Various cyclization methods to eventually build up pyrrole rings in the tetraheterocyclic systems have been tested and used to prepare SN4, SN4'', and benzannulated SN4' derivatives. Among them, the Buchwald–Hartwig amination of brominated precursors, the thermolysis of azide precursors, and a Cadogan cyclization of nitro-substituted precursors were successfully applied. The various heteroatom sequences ‘SSNS*’*, ‘SNNS*’*, and benzannulated ‘NSSN*’* allowed for evaluation of structure–property relationships relative to sulfur analogue tetrathienoacene (‘SSSS*’*). In line with the results for the whole series of *S,N*-heteroacenes, we found that replacement of sulfur by nitrogen atoms in the tetracyclic systems leads to a red-shift in absorption, a decrease in oxidation potential and energy gap. On the other hand, the replacement of a thiophene ring by benzene leads to the opposite effects. The basic SN4 and SN4'' frameworks represent novel core molecules for further functionalization, for example with terminal acceptor groups, leading to highly absorbing dye molecules for application in organic solar cells.

## Supporting Information

File 1 Synthetic procedures, ^1^H, ^13^C NMR, HRMS, and UV–vis spectra as well as cyclic voltammograms.

File 2 Crystallographic data.
