# Supplementary material for: Synthesis and characterization of S,N-heterotetracenes
Source: Beilstein J Org Chem. 2020 Oct 26;16:2636–44. doi: 10.3762/bjoc.16.214 (PMC7607432; doi:10.3762/bjoc.16.214)
Supplement: File 1 — Synthetic procedures, 1H, 13C NMR, HRMS, and UV–vis spectra as well as cyclic voltammograms. [file Beilstein_J_Org_Chem-16-2636-s001.pdf]

BEILSTEIN JOURNAL OF ORGANIC CHEMISTRY

Supporting Information

for

Synthesis and characterization of *S,N*-heterotetracenes

Astrid Vogt, Florian Henne, Christoph Wetzel, Elena Mena-Osteritz and Peter Bäuerle

Beilstein J. Org. Chem. **2020,** *16,* 2636–2644. doi:10.3762/bjoc.16.214

Synthetic procedures, ^{1}H, ^{13}C NMR, HRMS, and UV–vis spectra as well as cyclic voltammograms

License and Terms: This is a supporting information file under the terms of the Creative Commons Attribution License (https://creativecommons.org/licenses/by/4.0). Please note that the reuse, redistribution and reproduction in particular requires that the authors and source are credited.
The license is subject to the *Beilstein Journal of Organic Chemistry* terms and conditions: (https://www.beilstein-journals.org/bjoc)

Table of Content

	page
1. Materials and methods	S2
2 Synthetic procedures and analytical data	S4
3. ^{1}H-, ^{13}C-NMR spectra, high resolution mass spectra	S18
4. Optical spectra and cyclic voltammograms of target heteroacenes **13**, **19**, **22**	S23
5. Single crystal X-ray structure analysis of thienopyrrole **25**	S24
6. References	S26

1. Materials and methods

Instruments and measurements. NMR spectra were recorded on a Bruker AMX 500 (^{13}C NMR: 125 MHz; ^{1}H NMR: 500 MHz) or an Avance 400 Spectrometer (^{13}C NMR: 100 MHz; ^{1}H NMR: 400 MHz). Chemical shift values (δ) are expressed in parts per million (ppm) using residual solvent protons (δ = 3.58 (THF-d_8), 5.32 (CD_2Cl_2), 2.50 (DMSO-d_6) and 7.26 ($CDCl_3$) for ^{1}H-NMR spectra and δ = 67.21 (THF-d_8), 53.84 (CD_2Cl_2), 39.52 (DMSO-d_6) and 77.16 ($CDCl_3$) for ^{13}C NMR spectra) as internal standard. Coupling constants *J* relate to proton-proton couplings. The splitting patterns are designated as follows: s (singlet), d (doublet), t (triplet), and m (multiplet). The assignments of the protons correspond to TT-α-*H* (α-proton of thienothiophene unit), TT-β-*H* (β-proton of thienothiophene unit), Th-α-*H* (α-proton of terminal thiophene unit), Th-β-*H* (β-proton of terminal thiophene unit), TP-α-*H* (α-proton of thienopyrrole unit), TP-β-*H* (ß-proton of thienopyrrole unit), Py-α-*H* (α-proton of pyrrole unit), Py-β-*H* (ß-proton of pyrrole unit), Ph-H (phenyl protons). Thin layer chromatography was carried out on aluminum plates, pre-coated with silica gel, Merck Si60 F_{254}. Preparative column chromatography was performed on glass columns packed with silica gel 60 M (Macherey-Nagel) particle size 0.04–0.063 mm or silica gel 60 (Macherey-Nagel) particle size 0.063-0.2 mm. Melting points were determined using a Mettler Toledo DSC 823e under Ar flow (heating rate 10 °C/min) or a Büchi Melting Point M-565 (not corrected). GC–MS measurements were performed on a Shimadzu GCMS-QP2010 SE. MALDI–TOF mass spectra were measured on Bruker Daltonik Reflex III and high resolution MALDI mass spectra were performed on a Bruker SolariX using trans-2-[3-(4-*tert*-butylphenyl)-2-methyl-2-propenylidene]malononitrile (DCTB) as matrix. CI mass spectra were recorded on a Finnigan MAT, SSQ-7000. EI mass spectra were recorded on a Varian Saturn 2000 GC–MS. High resolution APCI spectra were performed on a Bruker SolariX using acetonitrile as solvent. Elemental analyses were performed on an Elementar Vario EL.

Optical measurements in solution were carried out in 1 cm cuvettes with Merck Uvasol grade solvents. Absorption spectra were recorded on a Perkin Elmer Lambda 19 spectrometer and corrected fluorescence spectra were recorded on a Perkin Elmer LS 55 fluorescence spectrometer. Cyclic voltammetry experiments were performed with a computer-controlled Autolab PGSTAT30 potentiostat in a three-electrode single-compartment cell (3 mL). The platinum working electrode consisted of a platinum wire sealed in a soft glass tube with a surface of A = 0.785 mm^2, which was polished down to 0.25 µm with Buehler polishing paste prior to use to guarantee reproducible surfaces. The counter electrode consisted of a platinum wire

and the reference electrode was an Ag/AgCl reference electrode. All potentials were internally referenced to the ferrocene/ferricenium couple (Fc/Fc^+). For the measurements, concentrations of 10^{-3} M of the electroactive species were used in freshly distilled and deaerated dichloromethane purified with a Braun MB-SPS-800 and 0.1 M $(n\text{-Bu})_4NPF_6$ (Fluka; recrystallized twice from ethanol).

Single crystals of thienopyrrole **25** were analysed on a Bruker SMART APEX-II CCD diffracttometer (λ(Mo$K\alpha$)-radiation, graphite monochromator, ω and 4 scan mode) and corrected for absorption using the SADABS program [1]. The structures were solved by direct methods and refined by a full-matrix least squares technique on F^2 with anisotropic displacement parameters for non-hydrogen atoms. The hydrogen atoms were placed in calculated positions and refined within the riding model with fixed isotropic displacement parameters (U_{ISO}(H) ¼ 1.2Ueq(C)). All calculations were carried out using the SHELXL program package in Olex2 (v. 1.2.10) [2]. Crystallographic data have been deposited with the Cambridge Crystallographic Data Center: CCDC 2016738.

Materials. Toluene (Sigma Aldrich), THF (Carl Roth GmbH), DMF, dichloromethane, and diethyl ether (VWR) were dried and purified by a MB SPS-800 (MBraun). Petroleum ether, $ZnCl_2$ (dried), methanol, and ethyl acetate were purchased from VWR, chlorobenzene from Merck and distilled prior to use. Sodium hydroxide, potassium hydroxide, sodium *tert*-butoxide, sodium pyrophosphate, *n*-propylamine, *n*-propyl iodide, *n*-hexylamine, *N*-bromosuccinimide, triisopropylsilyl chloride, diisopropylamine, triethyl phosphite, quinoline, copper powder, thiophene-2-carbaldehyde, and tetrabutylammonium hydrogensulfate were purchased from Merck. Pd(dppf) $Cl_2{\cdot}CH_2Cl_2$, tetra-*n*-butylammonium fluoride trihydrate, trimethyltin chloride, 2,2'–bis(diphenylphosphino)-1,1'-binaphthyl (BINAP), $Pd(dba)_2$, $Pd_2(dba)_3$, methyl-2-azidoacetate, and 1-iodo-2-nitrobenzene **17** were purchased from Sigma Aldrich, *n*-BuLi (1.6 M in hexane) from Acros Organics. $Pd[PPh_3]_4$ [3], thieno[3,2-*b*]thiophene **1** [4], 2,3-dibromothiophene [5], tosylazide [6], and 2-bromo-3-nitrothiophene **15** [7] were internally synthesized according to literature-known procedures.

2. Synthetic procedures and analytical data

2,5-Dibromothieno[3,2-*b*]thiophene (2). Synthesis was analogous as described in [8]. Thieno[3,2-*b*]thiophene **1** (1.40 g, 10.0 mmol) was dissolved in dry DMF (20 mL) at 0 °C. *N*-Bromosuccinimde (3.56 g, 20.00 mmol) was added and the resulting solution was allowed to slowly warm to room temperature. After stirring for 17 h, the solution was poured into water (200 mL) and extracted with diethyl ether (2 × 50 mL). The combined organic phases were washed with water (100 mL) and dried over $MgSO_4$. After removing the solvent in vacuum, the residue was further purified by column chromatography (silica gel; petroleum ether) to yield 2,6-dibromothieno[3,2-*b*]thiophene **17** (2.71 g, 9.10 mmol, 91%) as a colourless solid. Mp 118.3-121.6 °C; ^{1}H NMR (400 MHz, $CDCl_3$, 20 °C) δ (ppm) 7.15 (s, 1H; TT-β-*H*); ^{13}C-NMR (100 MHz, $CDCl_3$, 20 °C) δ ppm 138.4, 121.8, 113.7; MS (EI): *m/z* (%): 298 (100) $[M]^{+}$. Analytical data is in accordance with literature data [8].

2-Bromo-5-(triisopropylsilyl)thieno[3,2-*b*]thiophene (3). Synthesis was analogous as described in [9]. 2,5-Dibromothieno[3,2-*b*]thiophene **2** (2.36 g, 7.92 mmol) was dissolved in dry THF (83 mL) at -78 °C. To this solution *n*-BuLi (4.95 mL, 7.92 mmol, 1.6 M in hexane) was added dropwise. After stirring for 15 min at –78 °C, triisopropylsilyl chloride (1.6 mL, 7.92 mmol) was added dropwise and the solution was stirred for 3 h at this temperature. The solution was allowed to slowly warm to room temperature overnight. *n*-Hexane (50 mL) and brine (50 mL) were added. The organic phase was collected and dried over $MgSO_4$. After removing the solvent in vacuum, the residue was further purified by column chromatography (flash-silica gel; petroleum ether) to yield 2-bromo-5-(triisopropylsilyl)thieno[3,2-*b*]thiophene **3** (2.05 g, 5.46 mmol, 69%) as a white solid. Mp 73.8-74.2 °C; ^{1}H-NMR (400 MHz, $CDCl_3$, 20 °C) δ (ppm) 7.26 (s, 1H; TT-α-*H*), 7.26 (s, 1H; TT-β-*H*), 1.30-1.41 (m, 3H; Si-$(CH)_3$), 1.11 (d, $^{3}J_{H,H}$ = 7.4 Hz, 18H; Si-$(CH)_3$-$(CH_3)_6$); ^{13}C-NMR (100 MHz, $CDCl_3$, 20 °C) δ (ppm) 143.0, 141.0, 137.0, 126.4, 122.0, 114.5, 18.7, 11.9; MS (EI): *m/z* (%): 376 (100) $[M]^{+}$. Analytical data is in accordance with literature data [9].

3-Bromo-5-(triisopropylsilyl)thieno[3,2-*b*]thiophene (4). Synthesis was analogous as described in [9]. To a solution of diisopropylamine (0.64 mL, 4.55 mmol) in dry THF (55 mL) at 0 °C was added *n*-BuLi (1.6 M in *n*-hexane, 2.40 mL, 3.84 mmol) under Ar atmosphere. After

stirring for 30 min at this temperature, the solution was cooled to –78 °C. 2-Bromo-5-(triisopropylsilyl)thieno[3,2-*b*]thiophene **3** (1.25 mg, 3.33 mmol) was dissolved in dry THF (7 mL) and then added to the reaction mixture within 5 sec. After 1.5 h, the solution was diluted with petroleum ether (50 mL) and with brine (30 mL). The organic layer was separated and dried over $MgSO_4$. After removing the solvent in vacuo, the residue was purified by column chromatography (silica gel, petroleum ether). 3-Bromo-5-(triisopropylsilyl)thieno[3,2-*b*]thiophene **4** (1.14 g, 3.04 mmol, 91%) was obtained as a white solid. Mp 73.3-75.2 °C; ^{1}H-NMR (400 MHz, $CDCl_3$, 20 °C) δ (ppm) 7.39 (s, 1H; TT-α-*H*), 7.29 (s, 1H; TT-β-*H*), 1.32-1.41 (m, 3H; Si-(C*H*)$_3$), 1.13 ppm (d, $^3J_{H,H}$ = 7.4 Hz, 18H; Si-(CH)$_3$-(C*H*$_3$)$_6$); ^{13}C-NMR (100 MHz, $CDCl_3$, 20 °C) δ (ppm) 145.4, 140.2, 139.5, 127.7, 124.7, 102.3, 18.6, 11.8; MS (EI): *m/z* (%): 376 (100) [*M*]$^+$. Analytical data is in accordance with literature data [9].

2,3-Dibromo-5-(triisopropylsilyl)-thieno[3,2-*b*]thiophene (5). Synthesis was analogous as described in [10]. 3-Bromo-5-(triisopropylsilyl)thieno[3,2-*b*]thiophene **4** (1.02 g, 2.72 mmol) was dissolved in DMF (100 mL) at 0 °C. *N*-Bromosuccinimide (532 mg, 2.99 mmol) was added and the solution was allowed to slowly warm to room temperature. After 17 h, diethyl ether (150 mL) and water (200 mL) were added. The aqueous phase was extracted with diethyl ether (2 × 150 mL). The combined organic phases were dried over $MgSO_4$. After removing the solvent in vacuo, the residue was further purified by column chromatography (flash-silica gel; petroleum ether) to afford (5,6-dibromothieno[3,2-*b*]thien-2-yl)triisopropylsilane **5** (1.14 g, 2.51 mmol, 92 %) as a white solid. Mp 78.8 °C (DSC); ^{1}H-NMR (400 MHz, CD_2Cl_2, 20 °C) δ (ppm) 7.36 (s, 1H; Th-β-*H*), 1.37 (m, 3H; Si-(C*H*)$_3$), 1.12 ppm (d, $^3J_{H,H}$ = 7.4 Hz, 18H; Si-(CH)$_3$-(C*H*$_3$)$_6$); ^{13}C-NMR (100 MHz, CD_2Cl_2, 20 °C) δ (ppm) 144.2, 138.7, 138.4, 127.8, 113.2, 106.8, 18.7, 12.2; MS (EI): *m/z* (%): 454 (75) [*M*]$^+$, 411 (100) [M-C_3H_7]$^+$, 369 (25) [M-2C_3H_7]$^+$; anal. calcd. (%) for $C_{15}H_{22}Br_2S_2Si$: C 39.65, H 4.88, S 14.11; found: C 39.61, H 4.88, S 14.25. Analytical data is in accordance with literature data [10].

2-(3-Bromothien-2-yl)-3-bromo-5-(triisopropylsilyl)thieno[3,2-*b*]thiophene (7). Derivative **7** has been first shown in [11], but no synthetic procedure was given. *N*-BuLi (0.43 mL, 0.69 mmol, 1.6 M in *n*-hexane) was added slowly to a solution of 2,3-dibromothiophene **6** (148 mg, 0,61 mmol) in diethyl ether (2 mL) at -78 °C. After stirring for 1 h at this temperature, $ZnCl_2$ (91 mg, 0.67 mmol) dissolved in THF (2.8 mL) was added dropwise. After stirring for 1 h at 0 °C, (5,6-dibromothieno[3,2-b]thien-2-yl)triisopropylsilane **5** (185 mg, 0.41 mmol) and

$Pd(dppf)Cl_2{\cdot}CH_2Cl_2$ (20 mg, 0,024 mmol) were added and the mixture was stirred for 24 h at 50 °C. After removing the solvent in vacuum, the crude product was further purified by column chromatography (flash silica gel, petroleum ether) to give the pure product **7** (153 mg, 0.29 mmol, 70%) as a white solid in 70% yield. Mp 146.9 °C (DSC); ^{1}H-NMR (400 MHz, THF-d_8, 20 °C) δ (ppm) 7.69 (d, $^3J_{H,H}$ = 5.4 Hz, 1H; Th-α-*H*), 7.62 (s, 1H, TT-β-*H*), 7.16 (d, $^3J_{H,H}$ = 5.4 Hz, 1H; Th-β-*H*), 1.38-1.49 (m, 3H; Si-(C*H*)$_3$), 1.17 (d, $^3J_{H,H}$ = 7.4 Hz, 18H; Si-(CH)$_3$-(C*H$_3$*)$_6$); ^{13}C-NMR (100 MHz, THF-d_8, 20 °C) δ (ppm) 146.4, 140.7, 139.5, 131.6, 131.5, 130.1, 129.6, 129.1 113.4, 105.1, 18.7, 12.5; MS (EI): *m/z* (%): 536 (90) [*M*]$^+$, 493 (70) [M-C_3H_7]$^+$, 458 (40) [M-Br]$^+$, 157 (100) [M-$C_{11}H_{10}Br_2S_2$]$^+$; anal. calcd. (%) for $C_{15}H_{22}Br_2S_2Si$: C 42.54, H 4.51, S 17.93; found: C 42.68, H 4.62, S 18.11.

4-Hexyl-6-(triisopropylsilyl)thieno[3,2-*b*]thieno[2',3':4,5]thieno[2,3-*d*]pyrrole (8). Derivative **8** has been first shown in [11], but no synthetic procedure was given. Dibromide **7** (155 mg, 0.29 mmol), sodium *tert*-butoxide (278 mg, 2.89 mmol), $Pd(dba)_2$ (17.0 mg, 0.03 mmol) and dppf (64.0 mg, 0.12 mmol) were stirred in dry and degassed toluene (10 mL) at room temperature. After 20 min, *n*-hexylamine (0.56 mL, 0.43 mmol) was added and the mixture was stirred for 18 h at 110 °C. Water (30 mL) was added to the cooled reaction mixture and the aqueous phase was extracted with diethyl ether (20 mL) and dichloromethane (2 × 15 mL). The solvents were removed under reduced pressure. The crude product was purified by column chromatography under exclusion of light (flash-silica gel; *n*-hexane/dichloromethane: 10/1). Pure TIPS-SN4 **8** was obtained as a white solid (119 mg, 0.25 mmol, 87%). Mp 88.8 °C (DSC); ^{1}H-NMR (400 MHz, THF-d_8, 20 °C) δ (ppm) 7.51 (s, 1H; TT-β-*H*), 7.19 (d, $^3J_{H,H}$ = 5.3 Hz, 1H; Th-α-*H*), 7.12 (d, $^3J_{H,H}$ = 5.3 Hz, 1H; Th-β-*H*), 4.38 (t, $^3J_{H,H}$ = 7.0 Hz, 2H; N-C*H$_2$*), 1.91-1.98 (m, 2H; N-CH$_2$-C*H$_2$*), 1.27-1.47 (m, 9H; N-(CH$_2$)$_2$-(CH$_2$)$_3$+Si-(CH)$_3$), 1.18 (d, $^3J_{H,H}$ = 7.4 Hz, 18H; Si-(CH)$_3$-(C*H$_3$*)$_6$), 0.85 (t, $^3J_{H,H}$ = 7.1 Hz, 3H; N-(CH$_2$)$_5$-C*H$_3$*); ^{13}C-NMR (100 MHz, THF-d_8, 20 °C) δ (ppm) 145.0, 141.3, 136.1, 133.2, 130.0, 129.6, 123.7, 118.0, 116.5, 111.7, 48.0, 32.2, 31.7, 27.2, 23.2, 18.9, 14.1, 12.6; MS (CI, 100 eV): *m/z* (%): 536 (10) [*M*+C_2H_5]$^+$, 476 (100) [*M*]$^+$, 432 (14) [*M*-C_3H_7]$^+$; anal. calcd. (%) for $C_{25}H_{37}NS_3Si$: C 63.10, H 7.84, N 2.94, S 20.22; found C 62.99, H 7.98, N 2.89, S 20.08.

4-Hexyl-thieno[3,2-*b*]thieno[2',3':4,5]thieno[2,3-*d*]pyrrole (9). Derivative **9** has been first shown in [11], but no synthetic procedure was given. Tetra-*n*-butylammonium fluoride trihydrate (444 mg, 1.41 mmol) was added to a solution of TIPS-SN4 **8** (112 mg, 0.24 mmol) in

THF (20 mL). The solution was stirred for 2 h under exclusion of light. Water (20 mL) was added and the aqueous phase was extracted with diethyl ether (20 mL) and dichloromethane (2 × 10 mL). The combined organic phases were dried over $MgSO_4$, filtered and the solvent was removed. Further purification by column chromatography (petroleum ether/dichloromethane: 4:1) gave Hex-SN4 **9** as a white solid (60 mg, 0.19 mmol, 80%). Mp 66.3 °C (DSC); ^{1}H-NMR (400 MHz, THF-d_8, 20 °C) δ (ppm) 7.39 (d, $^3J_{H,H}$ = 5.2 Hz, 1H; TT-*α-H*), 7.33 (d, $^3J_{H,H}$ = 5.2 Hz, 1H; TT-*β-H*), 7.19 (d, $^3J_{H,H}$ = 5.3 Hz, 1H; Th-*α-H*), 7.13 (d, $^3J_{H,H}$ = 5.3 Hz, 1H; Th-*β-H*), 4.36 (t, $^3J_{H,H}$ = 7.0 Hz, 2H; N-C*H₂*), 1.89–1.94 (m, 2H; N-CH_2-C*H*$_2$), 1.25–1.43 (m, 6H; N-$(CH_2)_2$-$(CH_2)_3$), 0.85 (t, $^3J_{H,H}$ = 7.1 Hz, 3H; N-$(CH_2)_5$-CH_3); ^{13}C-NMR (100 MHz, THF-d_8, 20 °C) δ (ppm) 145.2, 139.7, 136.7, 124.6, 124.5, 123.8, 122.1, 117.5, 116.8, 112.1, 51.1, 50.6, 48.4, 32.6, 32.1, 27.6, 23.5, 14.5; MS (CI, 100 eV): *m/z* (%): 348 (24) $[M+C_2H_4]^+$, 320 (100) $[M+H]^+$; HRMS (APCI): *m/z* $[M]^+$, calcd. for $C_{16}H_{17}NS_3$, 319.05176, found 319.05209, δ*m/m*= 1.03 ppm; anal. calcd. (%) for $C_{16}H_{17}NS_3$: C 60.15, H 5.36, N 4.38, S 30.11; found: C 60.27, H 5.36, N 4.32, S 30.06.

2-(3-Bromothien-2-yl)-5-(triisopropylsilyl)thieno[3,2-*b*]thiophene (10). In a dried Schlenk-tube *n*-BuLi (1.6 M in hexane, 2.81 mL, 4.50 mmol) was added dropwise to a stirred solution of 2,3-dibromothiophene **6** (0.99 g, 5.09 mmol) in dry ether (6 mL) at –78 °C. The resulting solution was stirred for 1 h at –78 °C. Then, a solution of $ZnCl_2$ (613 mg, 4.50 mmol) in dry THF (10 mL) was added. After stirring 1 h at 0 °C, thienothiophene **3** (0.78 g, 1.71 mmol) and $Pd(dppf)Cl_2{\cdot}CH_2Cl_2$ (167 mg, 0.20 mmol) were added. The mixture was heated to 50 °C. After 24 h the mixture was directly purified by column chromatography (flash-silica gel, petroleum ether) in order to obtain **10** (0.90 g, 1.96 mmol, 72%) as a colourless solid. Mp 72.7 °C (DSC); ^{1}H-NMR (400 MHz, $CDCl_3$, 20 °C) δ (ppm) 7.61 (d, $^6J_{H,H}$ = 0.6 Hz, 1H; TT-β-*H*), 7.35 (d, $^6J_{H,H}$ = 0.6 Hz, 1H; TT-β-*H*), 7.23 (d, $^3J_{H,H}$ = 5.4 Hz, 1H; Th-α-*H*), 7.04 (d, $^3J_{H,H}$ = 5.4 Hz, 1H; Th-β-*H*), 1.33-1.42 (m, 3H; Si-(C*H*)$_3$), 1.14 (d, $^3J_{H,H}$ = 7.4 Hz, 18H; Si-$(CH)_3$-$(CH_3)_6$); ^{13}C-NMR (100 MHz, $CDCl_3$, 20 °C) δ (ppm) 144.6, 141.4, 139.1, 136.6, 132.7, 132.2, 127.0, 125.1, 118.8, 108.3, 18.7, 11.9; MS (CI, 100 eV): *m/z* (%): 487 (23) $[M+C_2H_5]^+$, 449 (100) $[M]^+$, 413 (25) $[M-C_3H_7]^+$, 378 (12) $[M-Br]^+$, 157 (60) $[M-C_{10}H_4BrS_3]^+$; anal. calcd. (%) for $C_{19}H_{25}BrS_3Si$: C 49.87, H 5.51; found C 50.02, H 5.45.

2-(3-Azidothien-2-yl)-5-(triisopropylsilyl)thieno[3,2-*b*]thiophene (11). *N*-BuLi (1.6 M in hexane, 0.61 mL, 0.97 mmol) was added dropwise to a solution of **10** (405 mg, 0.89 mmol) in 5 mL dry diethyl ether at -78 °C and the resulting mixture was stirred for 1 h at this temperature. A

solution of tosylazide (192 mg, 0.97 mmol) in dry diethyl ether (3 mL) was added slowly. After stirring for 5 h at –78 °C, the solution was allowed to warm to -30 °C before diethyl ether (5 mL) and a solution of sodium pyrophosphate (235 mg, 0.89 mmol) in water (15 mL) were added. The reaction mixture was stirred overnight at 0 °C and extracted with diethyl ether (15 mL) and dichloromethane (2 × 15 mL). Finally, azide **11** (342 mg, 0.81 mmol, 92%) was purified by column chromatography (short column, silica gel, petroleum ether). The compound readily decomposed. Mp 89.0 °C (DSC); ^{1}H-NMR (500 MHz THF-d$_8$, 20 °C) δ (ppm) 7.54 (d, $^{6}J_{H,H}$ = 0.6 Hz, 1H; TT-β-*H*), 7.45 (d, $^{6}J_{H,H}$ = 0.6 Hz, 1H; TT-β-*H*), 7.43 (d, $^{3}J_{H,H}$ = 5.5 Hz, 1H; Th-α-*H*), 7.10 (d, $^{3}J_{H,H}$ = 5.5 Hz, 1H; Th-β-*H*), 1.36-1.44 (m, 3H; Si-(C*H*)$_3$), 1.16 (d, $^{3}J_{H,H}$ = 7.4 Hz, 18H; Si-(CH)$_3$-(C*H$_3$*)$_6$); ^{13}C-NMR (125 MHz, THF-d$_8$, 20 °C) δ (ppm) 145.6, 141.6, 138.2, 137.3, 132.8, 128.1, 125.3, 123.1, 121.6, 117.3, 18.8, 12.6; MS (MALDI): *m/z* calcd. for $C_{19}H_{25}NS_3Si$ (M^+-N_2) 391.1 found 391.0, 782.1 (2M^+-2N_2); anal. calcd. (%) for $C_{19}H_{25}N_3S_3Si$: C 54.37, H 6.00, N 10.01, S 22.92; found C 54.20, H 5.88, N 9.75, S 23.12.

4-*H*-6-(Triisopropylsilyl)thieno[3,2-*b*]thieno[2',3':4,5]thieno[2,3-*d*]pyrrole (12). A solution of azide **11** (263 mg, 0.63 mmol) in dry chlorobenzene (20 mL) was added slowly to dry chlorobenzene (13 mL) at 120 °C. The solution was stirred for 35 minutes at 135 °C before the solvent was evaporated under vacuum. The residue was purified by column chromatography (flash-silica gel; petroleum ether/dichloromethane 7:3) in order to isolate pure **12** (113 mg, 0.29 mmol, 46%) as a white solid. Mp 104.2 °C (DSC); ^{1}H-NMR (500 MHz, THF-d$_8$, 20 °C) δ (ppm) 10.98 (s, 1H; N-*H*), 7.48 (s, 1H; TT-β-*H*), 7.17 (d, $^{3}J_{H,H}$ = 5.3 Hz, 1H; Th-α-*H*), 7.05 (d, $^{3}J_{H,H}$ = 5.3 Hz, 1H; Th-β-*H*), 1.36-1.45 (m, 3H; Si-(C*H*)$_3$), 1.17 (d, $^{3}J_{H,H}$ = 7.4 Hz, 18H; Si-(CH)$_3$-(C*H$_3$*)$_6$); ^{13}C-NMR (100 MHz, THF-d$_8$, 20 °C) δ (ppm) 144.1, 141.2, 135.1, 132.8, 130.4, 130.0, 123.6, 119.2, 117.6, 112.8, 18.9, 12.6; MS (MALDI): *m/z* calcd. for $C_{19}H_{25}NS_3Si$ (M^+) 391.1 found 391.1; anal. calcd. (%) for $C_{19}H_{25}NS_3Si$: C 58.26, H 6.43, N 3.58, S 24.56; found C 58.49, H 6.56, N 3.49, S 24.76.

4-*H*-Thieno[3,2-*b*]thieno[2',3':4,5]thieno[2,3-*d*]pyrrole (13). A solution of **12** (60 mg, 0.15 mmol) and TBAF (1.45 g, 2.60 mmol) in THF (50 mL) was stirred at room temperature under exclusion of light. After 18 h, the solvent was evaporated and the residue was purified by column chromatography (silica gel, petroleum ether/dichloromethane 2:1). Pure *H*-SN4 **13** (36 mg, 0.15 mmol, 99%) was obtained as a colourless solid. Mp 186.3 °C (DSC); ^{1}H-NMR (400 MHz, THF-d$_8$, 20 °C) δ (ppm) 10.98 (s, 1H; N-*H*), 7.33 (d, $^{3}J_{H,H}$ = 5.2 Hz, 1H; TT-α-*H*), 7.30 (d, $^{3}J_{H,H}$ = 5.1

Hz, 1H; TT-β-*H*), 7.16 (d, $^3J_{H,H}$ = 5.3 Hz, 1H; Th-α-*H*), 7.05 (d, $^3J_{H,H}$ = 5.3 Hz, 1H; Th-β-*H*); ^{13}C-NMR (125 MHz, THF-d_8, 20 °C) δ (ppm) 144.3, 139.6, 135.8, 125.4, 124.3, 123.7, 122.1, 118.6, 117.9, 113.1; MS (CI, 100 eV): *m/z* (%): 264 (11) [*M*+C_2H_5]$^+$, 236, HRMS (MALDI): m/z [M+H$^+$], calcd. for $C_{10}H_5NS_2$, 235.96569, found 235.96573, δm/m = 0.17 ppm; anal. calcd. (%) for $C_{10}H_5NS_3$: C 51.04, H 2.14, N 5.95, S 40.87; found C 51.23, H 1.98, N 5.82, S 40.78.

2-Trimethylstannyl-thieno[3,2-*b*]thiophene (14). Thieno[3,2-*b*]thiophene **1** (300 mg, 2.14 mmol) in 10.0 mL dry THF was put at -78°C under argon atmosphere and *n*-BuLi (1.6 M in hexane, 1.40 mL, 2.25 mmol) was slowly added over 30 min. Then, the mixture was stirred at -78°C for 2 h. Trimethylstannyl chloride (874 mg, 4.39 mmol) was dissolved in THF (3.5 mL) and efficiently added at –78 °C. The reaction mixture was removed from cooling and slowly warmed to rt. Saturated sodium bicarbonate solution (100 mL) was added and the mixture extracted three times with dichloromethane (50 mL each), dried over magnesium sulfate and the solvents evaporated. Stannyl **14** (562 mg, 1.85 mmol, 87%) was isolated as a dark brown viscous oil and used without further purification. ^{1}H-NMR (400 MHz, $CDCl_3$, 20°C) δ (ppm) 7.31 (d, $^3J_{H,H}$ = 5.2Hz, 1H, TT-α-H), 7.25 (s, 1H, TT-β-H), 7.21 (dd, $^3J_{H,H}$ = 5.2, 0.7Hz, 1H, TT-β-H), 0.38 (s, 9H, Sn-$(CH_3)_3$); ppm; ^{13}C-NMR (100 MHz, $CDCl_3$, 20°C) δ (ppm) 145.3, 129.7, 127.4, 126.5, 119.2, 118.0, -8.1 ppm. The analytical data is in accordance with literature [10].

2-(3-Nitrothien-2-yl)-thieno[3,2-*b*]thiophene (16). Stannyl **14** (468 mg, 1.54 mmol) and 2-bromo-3-nitrothiophene **15** (707 mg, 3.40 mmol) were dissolved under argon atmosphere in-to well degassed DMF (8.0 mL) and 5 mol % tetrakis(triphenylphosphine)palladium(0) (89 mg, 77 µmol) were added. The reaction mixture was warmed in a sealed vessel for 48 h at 80 °C. Water (100 mL) was added and the mixture extracted three times with dichloromethane (100 mL each), dried over magnesium sulfate, the solvents stripped off and the residue purified by column chromatography (silica gel, petroleum ether/ethyl acetate 5:1). Pure thienothiophene **16** (266 mg, 0.99 mmol, 64%) was obtained as fine crystalline orange needles. Mp 123-124°C, ^{1}H-NMR (400 MHz, $CDCl_3$, 20°C) δ (ppm) 7.78 (s, 1H, Th-β-H), 7.65 (d, $^3J_{H,H}$ = 5.8Hz, 1H, Th-α-H), 7.50 (d, $^3J_{H,H}$ = 5.3Hz, 1H, Th-β-H), 7.28 (d, $^3J_{H,H}$ = 5.3Hz, 1H, Th-β-H), 7.23 (d, $^3J_{H,H}$ = 5.7Hz, 1H, Th-α-H); ^{13}C-NMR (100 MHz, $CDCl_3$, 20°C) δ (ppm) 142.4, 142.2, 139.5, 138.9, 132.3, 129.8, 125.9, 124.0, 123.1, 119.5; MS (CI, 100 eV): *m/z* (%): 296 (10) [*M*+C_2H_5]$^+$, 268 (100) [*M*]$^+$; anal. calcd. (%) for $C_{10}H_5NO_2S_3$: C 44.93, H 1.89, N 5.24, S 35.98; found: C 44.70, H 1.65, N 5.10, S 35.78.

4*H*-Thieno[3,2-*b*]thieno[2',3':4,5]thieno[2,3-*d*]pyrrole (13). Nitrothiophene **16** (50.0 mg, 0.19 mmol) was heated in degassed triethyl phosphite (2 mL) under argon atmosphere in a sealed vessel for 5 h at 160 °C. After cooling to rt, ethyl acetate (10 mL) and 6 N hydrochloric acid (2.0 mL) were added and the mixture stirred for 2 h. The aqueous phase was extracted twice with 50 mL ethyl acetate each, dried over magnesium sulfate and the solvent evaporated. The residue was purified by column chromatography (flash silica gel, petroleum ether/ ethyl acetate 5:1). Pure *H*-SN4 **13** (7.0 mg, 0.03 mmol, 16%) was obtained as a colorless solid. The analytical data is in accordance with the product obtained via the azide route (vide supra).

2-(Nitrophen-2-yl)-thieno[3,2-*b*]thiophene (18). Stannyl **14** (907 mg, 3.00 mmol) and 1-iodo-2-nitrobenzene **17 (**1.1 g, 4.5 mMol) were dissolved in degassed and dry DMF (16 mL) and 5 mol% tetrakis(triphenylphosphine)palladium(0) (173 mg, 0.150 mmol) were added. The reaction mixture was heated to 80 °C and stirred for 48 h. Water (150 mL) was added and the mixture extracted three times with dichloromethane (100 mL each), dried over magnesium sulfate, the solvents stripped off and the residue purified by column chromatography (flash silica gel, petroleum ether/ethyl acetate 5:1). Pure thienothiophene **18** (675 mg, 2.58 mmol, 86%) was obtained as light yellow crystals. Mp 68-70 °C; ^{1}H-NMR (400 MHz, $CDCl_3$, 20 °C) δ (ppm) 7.74-7.78 (m, 1H, Ph-H), 7.59 (dd, $^3J_{H,H}$ = 5.1, 0.9Hz, 2H, Ph-H), 7.49 (dd, $^3J_{H,H}$ = 8.1, 4.4Hz, 1H, Ph-H), 7.41 (d, $^3J_{H,H}$ = 5.3Hz, 1H, Th-α-H), 7.26-7.27 (m, 2H, Th-β-H); ^{13}C-NMR (100 MHz, $CDCl_3$, 20°C) δ (ppm) 149.6, 140.3, 139.7, 138.7, 132.5, 132.1, 129.0, 128.6, 128.1, 124.1, 119.6, 119.5; anal. calcd. (%) for $C_{12}H_7NO_2S_2$: C 55.16, H 2.70, N 5.36, S 24.54; found: C 55.06, H 2.69, N 5.31, S 24.45; HRMS (APCI): *m/z* [M$^+$], calcd. for $C_{12}H_7NO_2S_2$, 261.99910, found 261.99927, δ*m/m*= 0.65 ppm.

9*H*-Thieno[2‘,3‘:4,5]thieno[3,2-*b*]indole (19). Nitrobenzene **18** (100 mg, 0.38 mmol) was heated in degassed triethyl phosphite (2 mL) under argon atmosphere in a sealed vessel for 5 h at 160 °C. After cooling to rt, ethyl acetate (10 mL) and 6 N hydrochloric acid (2.0 mL) were added and the mixture stirred for 2 h. The aqueous phase was extracted twice with ethyl acetate (50 mL each), dried over magnesium sulfate and the solvent evaporated. The residue was purified by column chromatography (flash silica gel, petroleum ether/ethyl acetate 5:1). Pure indole **19** (51 mg, 0.22 mmol, 58%) was obtained as a colorless solid. Mp 225-226°C; ^{1}H-NMR (400 MHz, DMSO-d_6, 20 °C) δ (ppm) 11.86 (s, 1H, NH), 7.70-7.74 (m, 1H, Ph-H), 7.67 (d,

$^3J_{H,H}$ = 5.2 Hz, 1H, Th-α-H), 7.53 (d, $^3J_{H,H}$ = 5.2 Hz, 1H, Th-β-H), 7.51-7.54 (m, 1H, Ph-H), 7.20-7.24 (m, 1H, Ph-H), 7.10-7.14 (m, 1H, Ph-H); ^{13}C-NMR (100 MHz, DMSO-d$_6$, 20°C) δ (ppm) 141.0, 140.1, 134.7, 126.8, 122.3, 121.7, 119.4, 118.1, 117.3, 112.5; HRMS (APCI) m/z: [M$^+$]: calcd for $C_{12}H_7NS_2$ 227.99471, found 227.99454, δ*m/m* = 0.75 ppm.

2,6-Bis(trimethylstannyl)thieno[3,2-*b*]thiophene (20). Thieno[3,2-*b*]thiophene **1** (500 mg, 3.57 mmol) in dry THF (25 mL) was put at -78°C into a three-necked flask under argon atmosphere and *n*-BuLi (1.6 M in hexane, 4.68 mL, 7.49 mmol) was slowly added over 30 min. Then, the mixture was stirred at –78 °C for 2 h. Trimethylstannyl chloride (1.49 g, 7.49 mmol) was dissolved in THF (7.5 mL) and efficiently added at –78 °C. The reaction mixture was removed from cooling and slowly warmed to rt. Saturated sodium bicarbonate solution (150 mL) was added and the mixture extracted three times with dichloromethane (100 mL each), dried over magnesium sulfate and the solvents evaporated. Distannyl **20** (1.13 g, 2.42mmol, 68%) was isolated as a dark brown viscous oil and used without further purification. ^{1}H-NMR (400 MHz, $CDCl_3$, 20°C) δ (ppm) 7.27 (s, 2H, TT-β-H), 0.39 (s, 18H, Sn-$(CH_3)_3$); ^{13}C-NMR (100 MHz, $CDCl_3$, 20°C) δ (ppm) 147.6, 141.3, 126,2, 8.1. The analytical data is in accordance with literature [11].

2,6-Bis(nitrophen-2-yl)-thieno[3,2-*b*]thiophene (21). Distannyl **20** (894 mg 1,92 mmol) and 1-iodo-2-nitrobenzene **17 (**1.05 g 4.22 mmol) were dissolved in degassed, dry DMF (20 mL) and 5 mol % tetrakis(triphenylphosphine) palladium(0) (111 mg, 96 µmol) were added. The reaction mixture was heated to 80 °C and stirred for 48 h. Thienothiophene **21** precipitated during the reaction and was sucked off and washed with methanol. Pure **21** (631 mg, 1.65 mmol, 86%) was obtained as yellow crystals. Mp 269-270 °C; ^{1}H-NMR (400 MHz, DMSO-d$_6$, 100°C) δ (ppm) 7.98 (d, $^3J_{H,H}$ = 8.0 Hz, 2H, Ph-H), 7.74-7.80 (m ,4H, Ph-H), 7.66-7.69 (m, 2H, Ph-H), 7.56 (s, 2H, Th-β-H); ^{13}C-NMR (100 MHz, DMSO-d$_6$, 100°C) δ (ppm) 148.7, 139.4, 138.4, 132.1; 131.7, 129.4, 126.7, 123.5, 119.7; anal. calcd (%) for $C_{18}H_{10}N_2O_4S_2$: C 56.54, H 2.64, N 7.33, S 16.77; found: C 56.23, H 2.50, N 7.28, S 16.57.

6,12-Dihydroindolo[2'',3'':4',5']thieno[2',3':4,5]thieno[3,2-b]indole (22). Nitrophenyl-substituted thienothiophene **21** (100 mg, 0.26 mmol) was heated in degassed triethyl phosphite (2 mL) under argon atmosphere in a sealed vessel for 5 h at 160 °C. After cooling to rt, ethyl acetate (10 mL) and 6N hydrochloric acid (2.0 mL) were added and the mixture stirred for 2 h. Indole **22** was precipitated by addition of methanol, filtered, and washed with methanol and

was obtained as light brown solid (32 mg, 0.10 mmol, 38%). Mp 453 °C (DSC); ^{1}H-NMR (400 MHz, DMSO-d$_6$, 75°C) δ (ppm) 11.96 (s, 2H, N-H), 7.78 (d, $^{3}J_{H,H}$ = 7.6 Hz, 2H, Ph-H), 7.54 (d, $^{3}J_{H,H}$ = 8.1Hz, 2H, Ph-H), 7.22-7.26 (m, 2H, Ph-H), 7.12-7.16 (m, 2H, Ph-H); ^{13}C-NMR (100 MHz, DMSO-d$_6$, 75°C) δ (ppm) 140.1, 135.7, 124.0, 122.2, 121.8, 119.0, 117.3, 116.5, 112.0; HRMS (APCI) m/z: [M$^+$]: calcd for $C_{18}H_{10}N_2S_2$ 318.02799, found 318.02775, δ*m/m* = 0.76 ppm.

Methyl-(Z)-2-azido-(3-thien-2-yl)acrylate (24). A solution of sodium methanolate was prepared by slowly adding sodium (1.67 g, 72.8 mmol) to absolute methanol (37 mL) under argon. The solution was cooled to -10 °C and thiophene-2-carbaldehyde **23** (1.70 mL, 18.2 mmol) and methyl-2-azido acetate (35.4 mL, 72.8 mmol) were slowly added dropwise at the same time. The mixture was stirred for 1 h at -10°C and 3 h at rt. Subsequently, ice water was added and the mixture extracted four times with diethyl ether (50 mL each). The combined organic phases were dried over sodium sulfate, the solvent removed under reduced pressure and the residue was purified by column chromatography (silica gel, petroleum ether/ethyl acetate 4:1). Acrylate **24** (3.08 g, 14.7 mmol, 81%) was obtained as a yellow oil. ^{1}H-NMR (400 MHz, $CDCl_3$, 20 °C) δ (ppm) = 7.50 (dd, $^{3}J_{H,H}$ = 5.1 Hz, $^{4}J_{H,H}$ = 1.2 Hz, 1H; Th-α-*H*), 7.32 (ddd, $^{3}J_{H,H}$ = 3.7 Hz, $^{4}J_{H,H}$ = 1.2 Hz, ^{4}J(H,H) = 0.6 Hz, 1H; Th-3-β-*H*), 7.17 (d, $^{4}J_{H,H}$ = 0.6 Hz, 1H; Alken-*H*), 7.07 (dd, $^{3}J_{H,H}$ = 5.1 Hz, $^{3}J_{H,H}$ = 3.7 Hz, 1H; Th-4-β-*H*), 3.90 (s, 3H; C*H*$_3$); ^{13}C-NMR (100 MHz, $CDCl_3$, 20 °C) δ (ppm) = 163.8, 136.6, 132.2, 130.7, 127.2, 122.5, 119.7, 52.9. The analytical data is in accordance with literature data [12].

Methyl-4*H*-thieno[3,2-*b*]pyrrole-5-carboxylate (25). A solution of acrylate **24** (3.08 g, 14.7 mmol) in dry toluene (100 mL) was slowly dropped under argon atmosphere into boiling toluene (135 mL). Subsequently, the reaction mixture was heated under reflux for another hour and then cooled to rt. After removal of the solvent in vacuum, the product was purified by column chromatography (silica gel, petroleum ether/ethyl acetate 4:1). Pure indole **19** (51 mg, 0.22 mmol, 58%) was obtained as a colorless solid. Carboxylate **25** (2.65 g, 14.6 mmol, 99%) was isolated as orange solid. Mp 150.2 °C (DSC); ^{1}H-NMR (400 MHz, $CDCl_3$, 20 °C) δ (ppm) = 9.51 (br s, 1H; N*H*), 7.33 (d, $^{3}J_{H,H}$ = 5.3 Hz, 1H; Th-α-*H*), 7.14 (dd, $^{4}J_{H,H}$ = 1.8 Hz, ^{5}J(H,H) = 0.6 Hz, 1H; Py-β-*H*), 6.96 (dd, $^{3}J_{H,H}$ = 5.3 Hz, ^{5}J(H,H) = 0.7 Hz, 1H; Th-β-*H*), 3.92 (s, 3H; O-C*H*$_3$); ^{13}C-NMR (100 MHz, $CDCl_3$, 20 °C) δ (ppm) = 162.4, 141.7, 129.7, 126.7, 124.9, 111.3, 107.8, 51.9; MS (EI): *m/z* (%): 181 (100) [M]$^+$. The analytical data is in accordance with literature data [12].

Methyl-4-propyl-4*H*-thieno[3,2-*b*]pyrrole-5-carboxylate (26). To a solution of carboxylate **25** (2.08 g, 11.5 mmol) and tetra-*n*-butylammonium hydrogensulfate (0.93 g, 2.8 mmol) in toluene (140 mL) a 50% aqueous sodium hydroxide solution (40 mL, 1.5 mol) was added and stirred for 15 min at rt. Then, 1-iodopropane (2.46 mL, 25.2 mmol) was dropped into the reaction mixture and was heated to 80 °C for 3 days. After cooling to rt, the solution was extracted twice with ethyl acetate (70 mL each) and dichloromethane (70 mL each), dried over sodium sulfate, the solvent removed under reduced pressure and the residue was purified by column chromatography (silica gel, petroleum ether/ethyl acetate 4:1). Thienopyrrole **26** (2.52 g, 11.3 mmol, 99%) was isolated as brownish oil. The product contained minor traces of the corresponding propyl ester, which was not separated. Bp 328 °C; ^{1}H-NMR (400 MHz, $CDCl_3$, 20 °C) δ (ppm) = 7.32 (d, $^3J_{H,H}$ = 5.4 Hz, 1H; Th-α-*H*), 7.18 (d, $^5J_{H,H}$ = 0.7 Hz, 1H; Py-β-*H*), 6.94 (dd, $^3J_{H,H}$ = 5.4 Hz, $^5J_{H,H}$ = 0.7 Hz, 1H; Th-β-*H*), 4.45 (t, $^3J_{H,H}$ = 7.1 Hz, 2H; N-CH_2), 3.86 (s, 3H; O-CH_3), 1.78-1.89 (m, 2H; N-CH_2-CH_2), 0.91 (t, $^3J_{H,H}$ = 7.4 Hz, 3H; CH_3); ^{13}C-NMR (100 MHz, $CDCl_3$, 20 °C) δ (ppm) = 162.1, 145.4, 129.2, 126.0, 121.9, 110.5, 109.4, 51.4, 49.2, 24.5, 11.4; MS (EI): *m/z* (%): 223 (100) $[M]^+$.

4-Propyl-4*H*-thieno[3,2-*b*]pyrrole-5-carboxylic acid (27). Carboxylate **26** (2.52 g, 11.3 mmol) was dissolved in a mixture of ethanol (46 mL), THF (18 mL) and water (12 mL) and potassium hydroxide (2.54 g, 45.2 mmol) added. The reaction mixture was heated to 50 °C for 15 h and afterwards the solvents removed under reduced pressure. Water was added and the solution acidified to pH 2 with 10% aqueous HCl-solution. A solid precipitated, which was filtered off, washed with water, and dried over orange-gel in an exsiccator. Thienopyrrole carbonic acid **27** (2.30 g, 11.0 mmol, 97%) was isolated a yellow solid. Mp 149.1 °C (DSC); ^{1}H-NMR (400 MHz, DMSO-d_6, 20 °C) δ (ppm) = 12.45 (br s, 1H, COO*H*), 7.54 (d, $^3J_{H,H}$ = 5.4 Hz, 1H; Th-α-*H*), 7.23 (dd, $^3J_{H,H}$ = 5.4 Hz, $^5J_{H,H}$ = 0.6 Hz, 1H; Th-β-*H*), 7.14 (d, $^5J_{H,H}$ = 0.6 Hz, 1H; Py-β-*H*), 4.45 (t, $^3J_{H,H}$ = 7.0 Hz, 2H; N-CH_2), 1.65-1.77 (m, 2H; N-CH_2-CH_2), 0.79 (t, $^3J_{H,H}$ = 7.4 Hz, 3H; CH_3); ^{13}C-NMR (100 MHz, DMSO-d_6, 20 °C) δ (ppm) = 162.3, 145.1, 129.3, 126.4, 120.8, 111.4, 109.1, 48.1, 24.0, 11.0; MS (CI, 70 eV): *m/z* (%): 210 (100) $[M+H]^+$; anal. calcd (%) for $C_{17}H_{13}NS_4$: C 57.40, H 5.30, N 6.69, S 15.32; found: C 57.67, H 5.35, N 6.69, S 15.27. The analytical data is in accordance with literature data [11].

4-Propyl-4*H*-thieno[3,2-*b*]pyrrole (28). A mixture of carbonic acid **27** (2.00 g, 9.56 mmol) and copper powder (425 mg, 6.69 mmol) in quinoline (28 mL) was heated to 260 °C for 45 min until gas evolution stopped. After cooling to rt, diethyl ether (100 mL) was added and the organic phase washed with 1M aqueous HCl solution (2 L). Thienopyrrole **28** (1.49 g, 9.03 mmol, 94%) was isolated as a colorless oil after column chromatography (flash silica gel, petroleum ether. Bp 278 °C; ^{1}H-NMR (400 MHz, THF-d$_8$, 20 °C) δ (ppm) = 7.03 (dd, $^3J_{H,H}$ = 5.3 Hz, $^6J_{H,H}$ = 1.2 Hz, 1H; Th-α-*H*), 6.93 (d, $^3J_{H,H}$ = 5.3 Hz, 1H; Th-β-*H*), 6.87 (dd, $^3J_{H,H}$ = 2.9 Hz, $^6J_{H,H}$ = 1.2 Hz, 1H; Py-α-*H*), 6.26 (d, $^3J_{H,H}$ = 2.9 Hz, 1H; Py-β-*H*), 3.99 (t, $^3J_{H,H}$ = 7.0 Hz, 2H; N-C*H*$_2$), 1.75-1.86 (m, 2H; N-CH$_2$-C*H*$_2$), 0.87 (t, $^3J_{H,H}$ = 7.4 Hz, 3H; C*H*$_3$); ^{13}C-NMR (100 MHz, THF-d$_8$, 20 °C) δ (ppm) = 141.0, 126.7, 124.2, 123.4, 111.0, 100.6, 50.8, 25.1, 11.8; MS (EI): *m/z* (%): 165 (100) [M]$^+$.

4-Propyl-2-(triisopropylsilyl)-4*H*-thieno[3,2-*b*]pyrrole (29). Thienopyrrole **28** (1.96 g, 11.9 mmol) was dissolved in dry THF (130 mL) under a stream of argon and cooled to -78 °C. Subsequently, *n*-BuLi (7.78 mL, 12.5 mmol, 1.6 M in *n*-hexane) was slowly dropped in and the reaction mixture was stirred for additional 15 min at –78 °C. Then, it was warmed to rt and stirred for one hour at this temperature. The reaction mixture was cooled to -78 °C and triisopropylsilyl chloride (2.64 mL, 12.5 mmol) was dropped in and slowly warmed to rt over 14 h. Water was added and the solution extracted twice with diethyl ether (100 mL each) and twice with dichloromethane (100 mL each). The combined organic phases were dried over sodium sulfate, the solvent stripped off and the product was isolated by column chromatography (flash silica gel, petroleum ether). TIPS-protected thienopyrrole **29** (3.50 g, 10.9 mmol, 92%) was obtained as colorless solid. Mp 53.9 °C (DSC); ^{1}H-NMR (400 MHz, CD$_2$Cl$_2$, 20 °C) δ (ppm) = 7.04 (d, $^5J_{H,H}$ = 0.7 Hz, 1H; Th-β-*H*), 6.90 (d, $^3J_{H,H}$ = 2.9 Hz, 1H; Py-α-*H*), 6.33 (dd, $^3J_{H,H}$ = 2.9 Hz, $^5J_{H,H}$ = 0.7 Hz, 1H; Py-β-*H*), 4.00 (t, $^3J_{H,H}$ = 7.0 Hz, 2H; N-C*H*$_2$), 1.80-1.91 (m, 2H; N-CH$_2$-C*H*$_2$), 1.29-1.43 (m, 3H; Si-(C*H*)$_3$), 1.14 (d, $^3J_{H,H}$ = 7.4 Hz, 18H; Si-(CH)$_3$-(C*H*$_3$)$_6$), 0.94 (t, $^3J_{H,H}$ = 7.4 Hz, 3H; CH$_2$-C*H*$_3$); ^{13}C-NMR (100 MHz, CD$_2$Cl$_2$, 20 °C) δ (ppm) = 142.7, 132.7, 129.1, 127.2, 118.4, 100.2, 50.8, 24.7, 19.0, 12.4, 11.8; MS (EI): *m/z* (%): 321 (100) [M]$^+$; anal. calcd (%) for $C_{18}H_{31}NSSi$: C 67.23, H 9.72, N 4.36, S 9.97; found: C 67.46, H 9.61, N 4.34, S 9.91.

5,6-Dibromo-4-propyl-2-(triisopropylsilyl)-4*H*-thieno[3,2-*b*]pyrrole (30). Thienopyrrole **29** (3.47 g, 10.8 mmol) was dissolved in THF (175 mL) and cooled to 0 °C. *N*-Bromosuccinimide (3.85 g, 21.6 mmol) was added in portions and the reaction mixture stirred for 14 h. Then, the

solvent was stripped off under reduced pressure and the product was filtered over a short column (flash silica gel, petroleum ether). Dibrominated thienopyrrole **30** (5.14 g, 10.7 mmol, 99%) was isolated as colorless solid. Mp 62.8 °C (DSC); ^{1}H-NMR (400 MHz, CD_2Cl_2, 20 °C) δ (ppm) = 7.04 (s, 1H; Th-β-*H*), 4.12 (t, $^3J_{H,H}$ = 7.1 Hz, 2H; N-C*H*$_2$), 1.75-1.86 (m, 2H; N-CH_2-C*H*$_2$), 1.28-1.42 (m, 3H; Si-(C*H*)$_3$), 1.12 (d, $^3J_{H,H}$ = 7.4 Hz, 18H; Si-(CH)$_3$-(C*H*$_3$)$_6$), 0.92 (t, $^3J_{H,H}$ = 7.4 Hz, 3H; CH_2-C*H*$_3$); ^{13}C-NMR (100 MHz, CD_2Cl_2, 20 °C) δ (ppm) = 141.7, 133.2, 128.4, 118.7, 109.6, 90.4, 50.5, 24.3, 18.9, 12.3, 11.5; MS (EI): *m/z* (%): 479 (100) [M]$^+$; anal. calcd (%) for $C_{18}H_{29}Br_2NSSi$: C 45.10, H 6.10, N 2.92, S 6.69; found: C 45.13, H 5.98, N 2.96, S 6.95.

6-Bromo-5-(3-bromothien-2-yl)-4-propyl-2-(triisopropylsilyl)-4*H*-thieno[3,2-*b*]pyrrole (31). 2,3-Dibromothiophene **6** (1.13 mL, 10.0 mmol) was dissolved in dry THF (50 mL) in a Schlenk tube under argon and cooled to –78 °C. Subsequently, *n*-BuLi (6.88 mL, 11.0 mmol, 1.6 M in *n*-hexane) was slowly dropped in and the reaction mixture was stirred for an additional hour at –78 °C. Then, anhydrous zinc chloride (1.46 g, 10.7 mmol) dissolved in dry THF (75 mL) was slowly dropped in. The reaction mixture was warmed to 0 °C and stirred for another hour at this temperature. Thienopyrrole **30** (3.20 g, 6.68 mmol) and [1,1′-bis(diphenylphosphino)ferrocene] dichloropalladium(II)·CH_2Cl_2 (382 mg, 0.468 mmol) were added and the reaction mixture was heated at 70 °C for 17 days. After removal of the solvent and column chromatography (flash silica gel, petroleum ether) thienopyrrole **31** (1.37 g, 2.44 mmol, 37%) was isolated as colorless solid. Mp 131.3 °C (DSC); ^{1}H-NMR (400 MHz, THF-d$_8$, 20 °C) δ (ppm) = 7.72 (d, $^3J_{H,H}$ = 5.4 Hz, 1H; Th-α-*H*), 7.24 (s, 1H; TP-β-*H*), 7.19 (d, $^3J_{H,H}$ = 5.4 Hz, 1H; Th-β-*H*), 3.90-4.10 (m, 2H; N-C*H*$_2$), 1.63-1.76 (m, 2H; N-CH_2-C*H*$_2$), 1.34-1.48 (m, 3H; Si-(C*H*)$_3$), 1.17 (d, $^3J_{H,H}$ = 7.4 Hz, 18H; Si-(CH)$_3$-(C*H*$_3$)$_6$), 0.79 (t, $^3J_{H,H}$ = 7.4 Hz, 3H; CH_2-C*H*$_3$); ^{13}C-NMR (100 MHz, CD_2Cl_2, 20 °C) δ (ppm) = 143.2, 135.2, 131.0, 129.6, 129.0, 128.2, 126.8, 119.1, 115.1, 91.8, 49.4, 24.5, 19.0, 12.3, 11.7; MS (EI): *m/z* (%): 561 (100) [M]$^+$; anal. calcd (%) for $C_{22}H_{31}Br_2NS_2Si$: C 47.06, H 5.56, N 2.49, S 11.42; found: C 47.06, H 5.48, N 2.61, S 11.37.

4,8-Dipropyl-2-(triisopropylsilyl)-4,8-dihydrothieno[2′,3′:4,5]pyrrolo[3,2-*b*]thieno[2,3-*d*]-pyrrole (32). Thienopyrrole **31** (1.00 g, 1.78 mmol), tris(dibenzylideneacetone) dipalladium (0) (82.0 mg, 0.09 mmol), 2,2′-bis(diphenylphosphino)-1,1′-binaphthyl (222 mg, 0.36 mmol) and sodium *tert*-butoxide (685 mg, 7.12 mmol) were dissolved in dry toluene (23 mL) in a Schlenk tube under argon and the solution was degassed with argon for 20 min. *n*-Propyl amine (0.22 mL, 2.7 mmol) was dropped in and the reaction mixture heated at 110 °C for 14 h. The

solvent was removed under reduced pressure and the residue subjected to column chromatography (flash silica gel, petroleum ether). Thienopyrrole **32** (501 mg, 1.09 mmol, 61%) was isolated as yellowish solid. Mp 97.3 °C (DSC). ^{1}H-NMR (400 MHz, THF-d_8, 20 °C) δ (ppm) = 7.22 (s, 1H; TP-3-β-*H*), 7.07 (d, $^{3}J_{H,H}$ = 5.2 Hz, 1H; TP-10-β-*H*), 7.03 (d, $^{3}J_{H,H}$ = 5.3 Hz, 1H; TP-α-*H*), 4.16-4.24 (m, 4H; N-C*H*$_2$, N-C*H*$_2$), 1.91-2.03 (m, 4H; N-CH$_2$-C*H*$_2$, N-CH$_2$-C*H*$_2$), 1.35-1.48 (m, 3H; Si-(C*H*)$_3$), 1.18 (d, $^{3}J_{H,H}$ = 7.4 Hz, 18H; Si-(CH)$_3$-(C*H*$_3$)$_6$), 0.91-0.99 (m, 6H; CH$_2$-C*H*$_3$, CH$_2$-C*H*$_3$); ^{13}C-NMR (100 MHz, THF-d_8, 20 °C) δ (ppm) = 146.2, 144.2, 128.5, 127.8, 126.8, 121.1, 119.9, 112.8, 112.2, 106.8, 49.8, 25.2, 19.3, 13.0, 12.0, 12.0; HRMS (APCI): *m/z* calcd for $C_{25}H_{38}N_2S_2Si$: 459.23184; found 459.23311 [M+H]$^+$ ($\delta m/m$ = 2.77 ppm).

4,8-Dipropyl-4,8-dihydrothieno[2',3':4,5]pyrrolo[3,2-*b*]thieno[2,3-*d*]pyrrole (33). Heteroacene **32** (35.0 mg, 0.08 mmol) was dissolved in THF (10 mL) under exclusion of light and tetra-*n*-butylammonium fluoride trihydrate (96 mg, 0.31 mmol) was added. The reaction mixture was stirred at r.t. over 14 h and filtered over a short column (silica gel, petroleum ether). Heterotetracene **33** (23.0 mg, 0.08 mmol, 98%) was isolated as a colorless solid. Mp 141.0 °C (DSC); ^{1}H-NMR (400 MHz, THF-d_8, 20 °C) δ (ppm) = 7.06 (d, $^{3}J_{H,H}$ = 5.2 Hz, 2H; TP-β-*H*), 7.02 (d, $^{3}J_{H,H}$ = 5.2 Hz, 2H; TP-α-*H*), 4.19 (t, $^{3}J_{H,H}$ = 6.9 Hz, 4H; N-C*H*$_2$), 1.90-2.01 (m, 4H; N-CH$_2$-C*H*$_2$), 0.93 (t, $^{3}J_{H,H}$ = 7.4 Hz, 6H; C*H*$_3$); ^{13}C-NMR (100 MHz, THF-d_8, 20 °C) δ (ppm) = 143.8, 126.9, 120.7, 112.1, 107.0, 49.7, 25.2, 12.0; HRMS (APCI): *m/z* calcd for $C_{16}H_{18}N_2S_2$: 303.09842; found 303.09853 [M+H]$^+$ ($\delta m/m$ = 0.36 ppm).

3. ^1H and ^{13}C-NMR spectra and high resolution mass spectra

4-Hexyl-thieno[3,2-*b*]thieno[2',3':4,5]thieno[2,3-*d*]pyrrole **9**

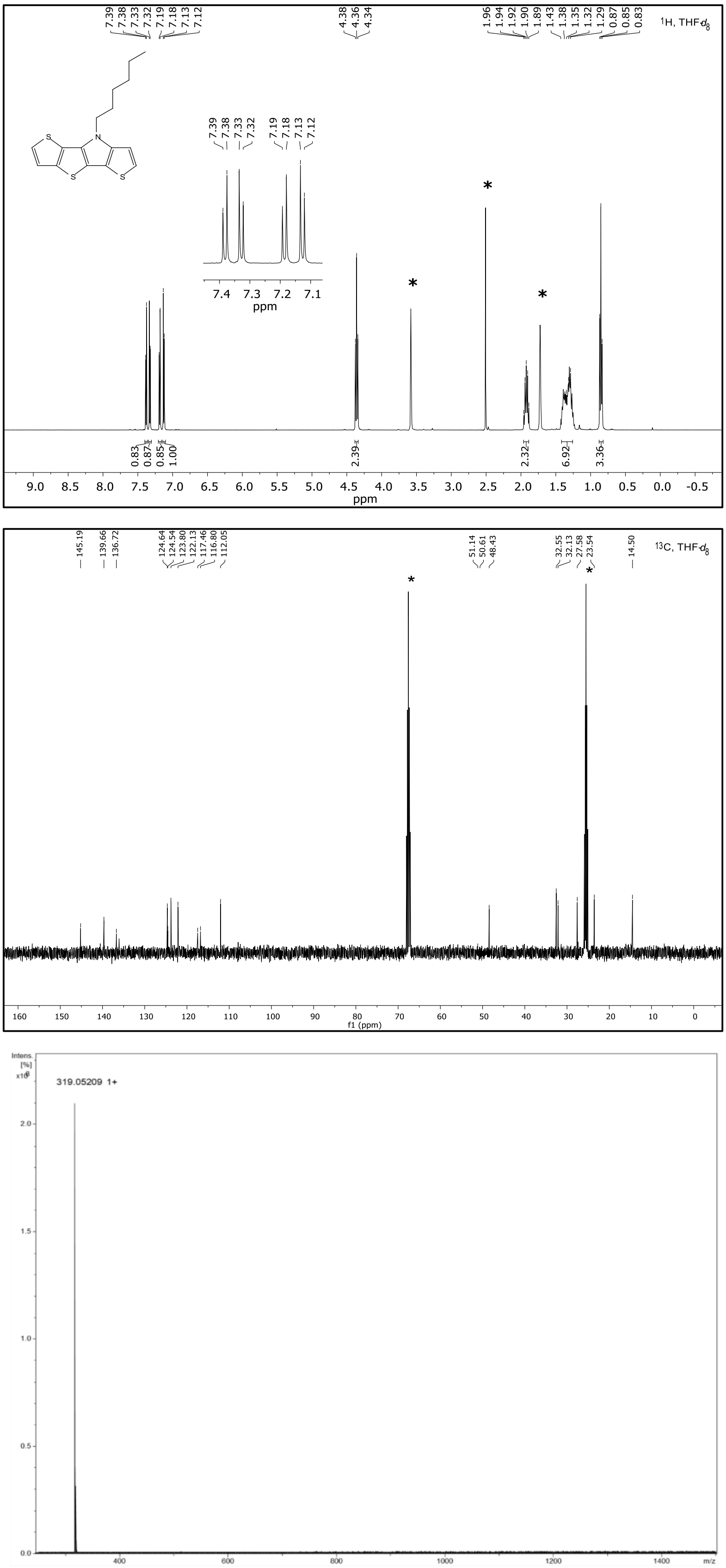

Figure S1: ^1H (top), ^{13}C-NMR spectrum (middle), and HRMS (bottom) of heterotetracene **9**.

4-*H*-Thieno[3,2-*b*]thieno[2',3':4,5]thieno[2,3-*d*]pyrrole **13**

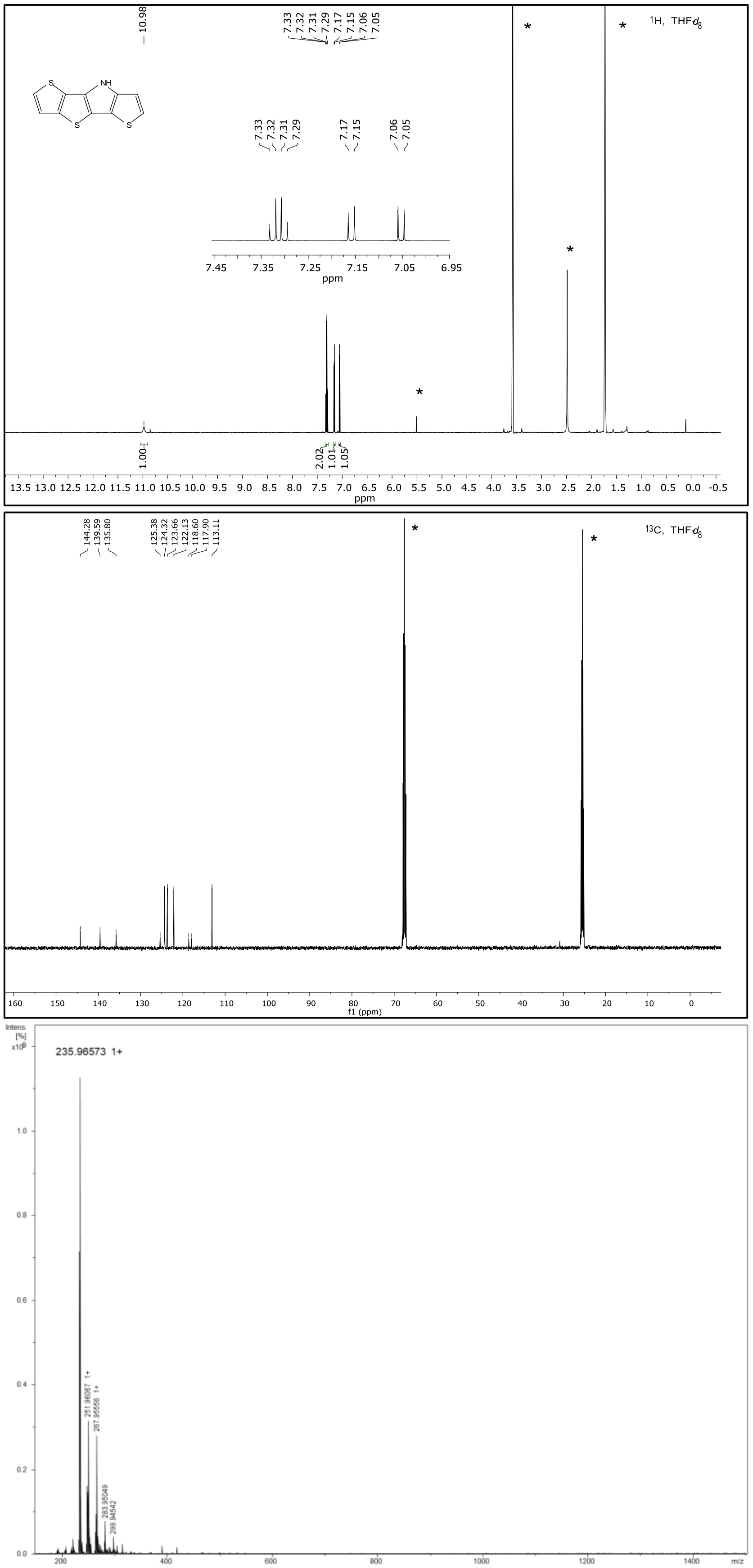

Figure S2: ^{1}H (top), ^{13}C-NMR spectrum (middle), and HRMS (bottom) of heterotetracene **13**.

9*H*-Thieno[2‘,3‘:4,5]thieno[3,2-*b*]indole **19**

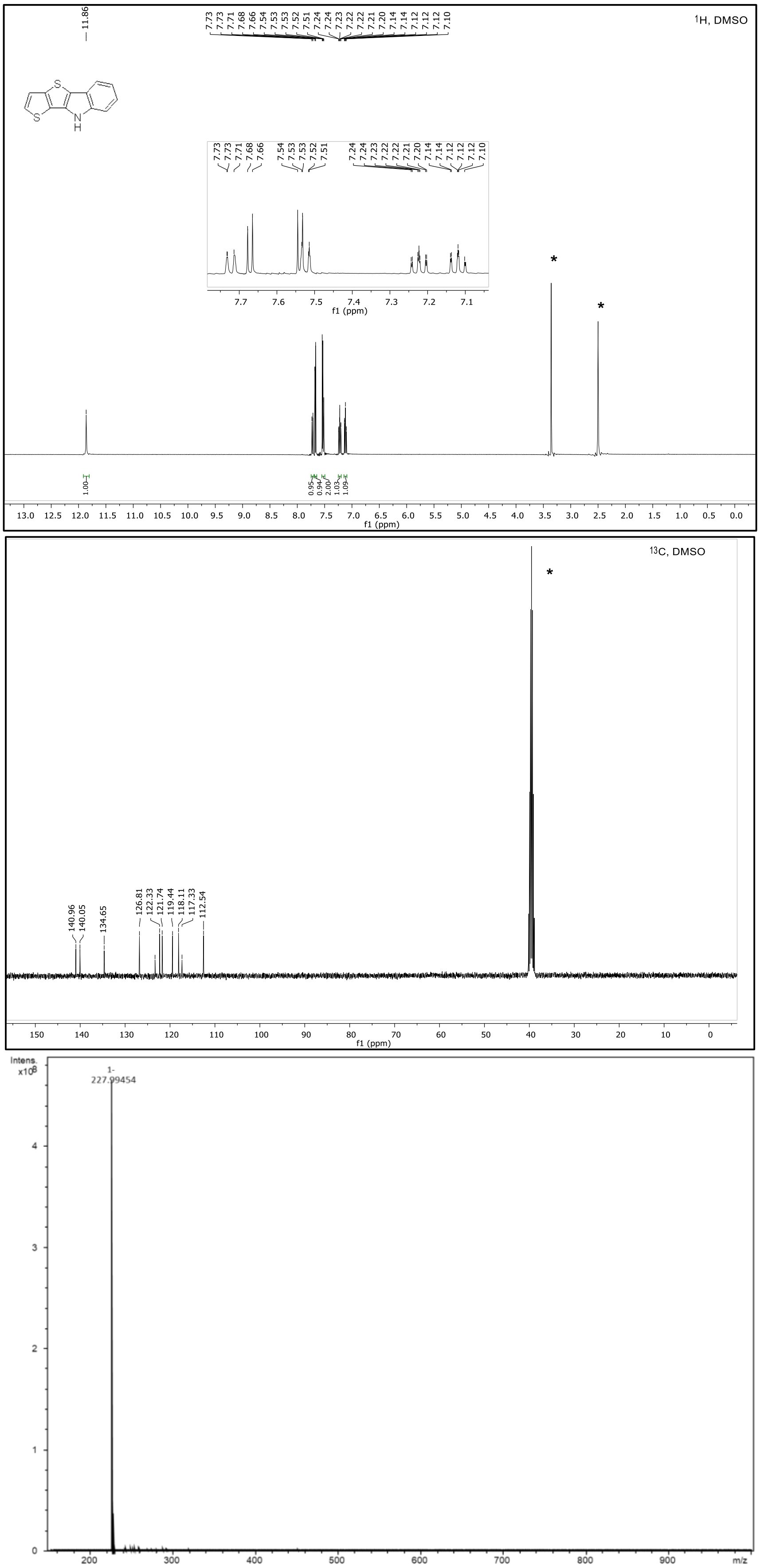

Figure S3: ^{1}H (top), ^{13}C-NMR spectrum (middle), and HRMS (bottom) of heterotetracene **19**.

6,12-Dihydroindolo[2'',3'':4',5']thieno[2',3':4,5]thieno[3,2-b]indole **22**.

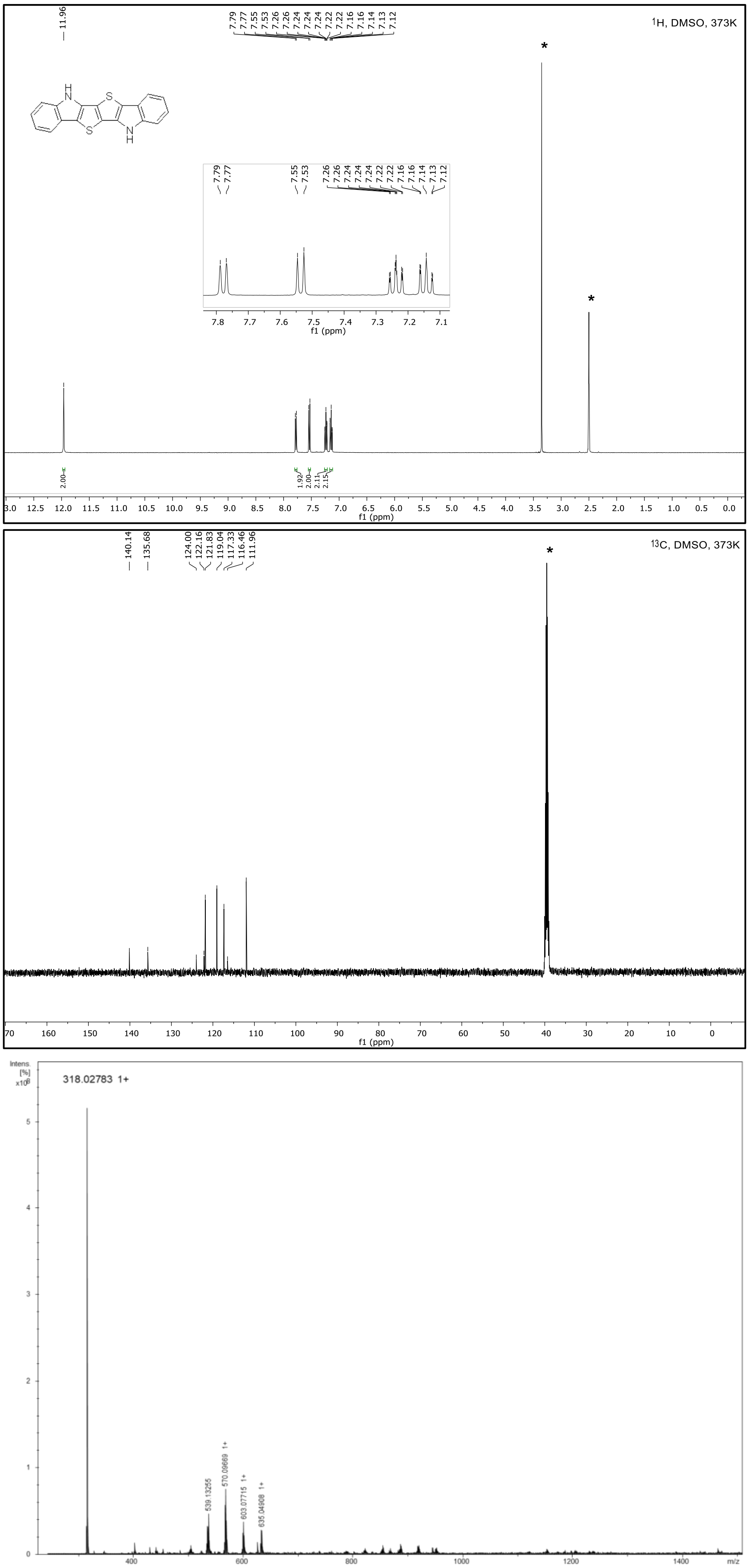

Figure S4: ^{1}H (top), ^{13}C-NMR spectrum (middle), and HRMS (bottom) of heterohexacene **22**.

4,8-Dipropyl-4,8-dihydrothieno[2’,3’:4,5]pyrrolo[3,2-*b*]thieno[2,3-*d*]pyrrole **33**

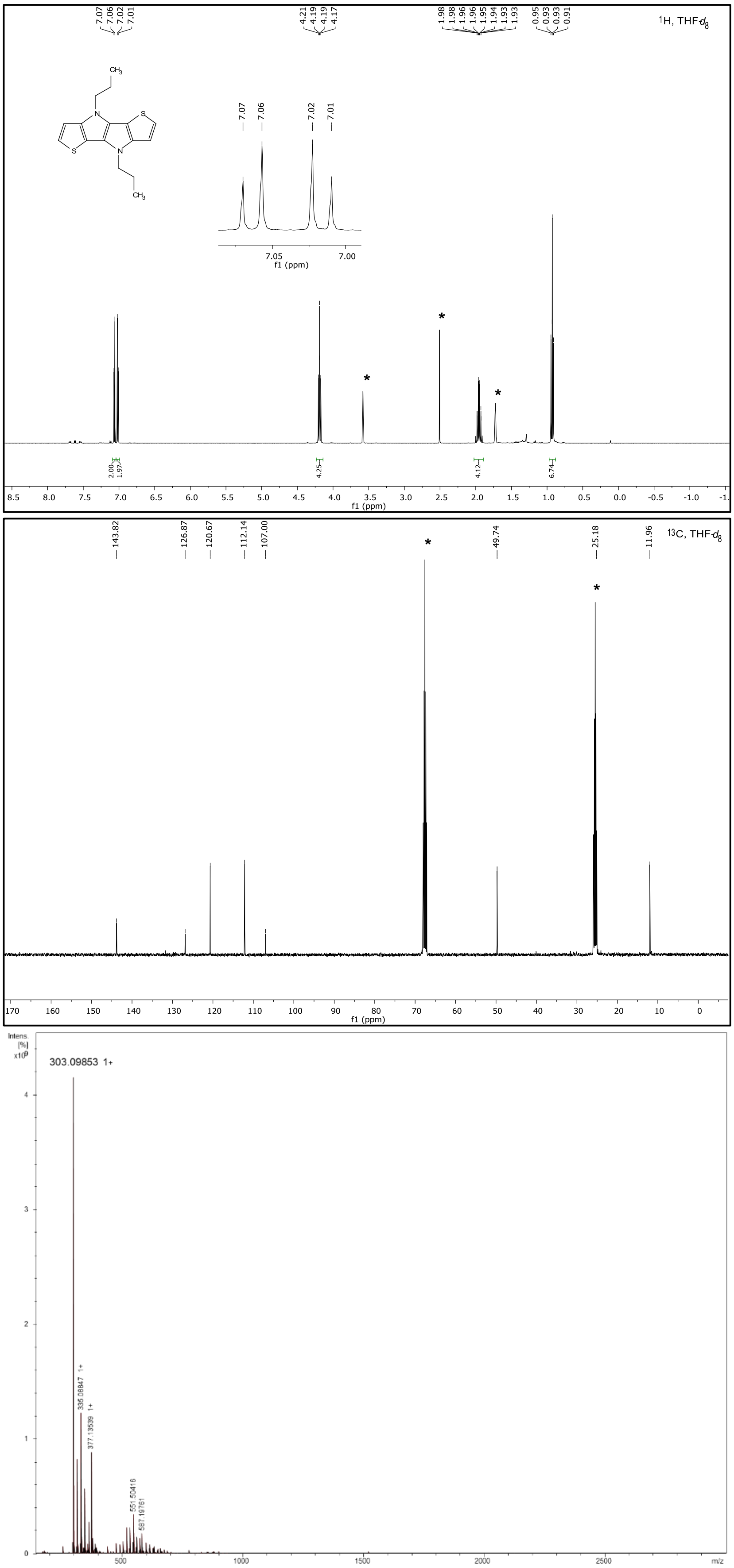

Figure S5: ^{1}H (top), ^{13}C-NMR spectrum (middle), and HRMS (bottom) of heterotetracene **33**

4. Optical spectra and cyclic voltammograms of target heteroacenes **13, 19, 22**

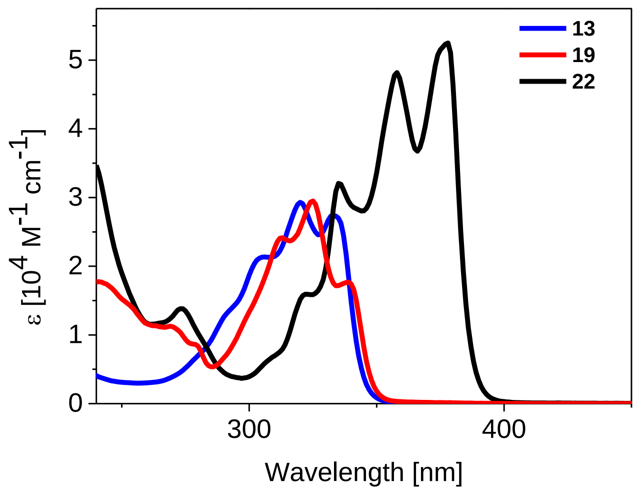

Figure S6: UV-vis spectra of *S,N*-heteroacenes **13**, **19**, and **22** measured in THF at 25 °C.

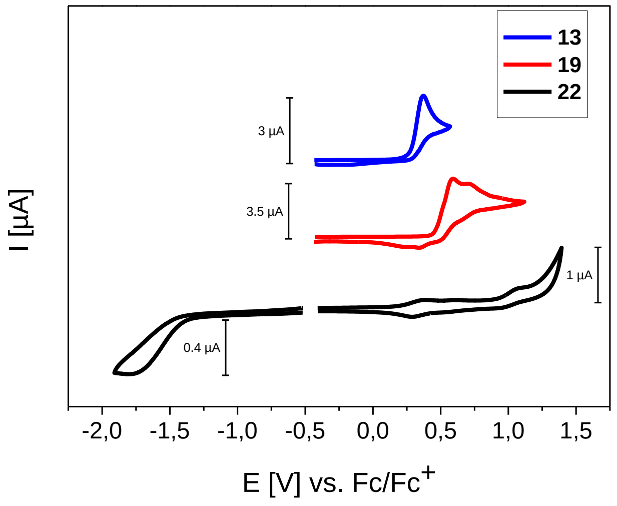

Figure S7: Cyclic voltammograms of *S,N*-heteroacenes **13**, **19**, and **22** measured in dichloromethane / $TBAPF_6$ (0.1 M), c= 10^{-3} M, scan rate = 100 mV s^{-1}.

5. Single crystal X-ray structure analysis

Single crystals suitable for X-ray structure analysis were obtained by slow evaporation of a chloroform solution of intermediate thienopyrrole **25**. The crystals belong to the monoclinic space group P 2_1/n. The unit cell (a = 11.0745(8) Å, b = 4.6972(3) Å, c = 15.6996(9) Å; α = 90°, β = 100.785(6)°, γ = 90°) contains four equivalent molecules arranging in a 2-fold screw axis symmetry. Bond lengths, angles, and torsion angles are compiled below in Tables S1-S4, atomic numbering in Figure S8. The heteroaromatic part of the molecule is almost coplanar with the ester group attached to the pyrrole ring. The molecules order in a herringbone fashion and furthermore interact in the (15 8 8) plane with an adjacent molecule via hydrogen bonds between the pyrrolic N-H and the C=O ester group (Figure S9, blue dotted lines). In the perpendicular direction to the (15 8 8) plane, the molecules interact via π–π stacking at distances as short as 3.437 Å. Single crystal X-ray structure analyses of thienopyrroles are so far only available for Fischer carbene complexes [15].

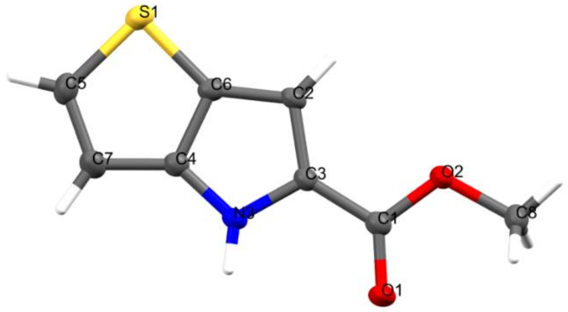

Figure S8: Molecular geometry of the asymmetric unit of **25** with heavy atom labels.

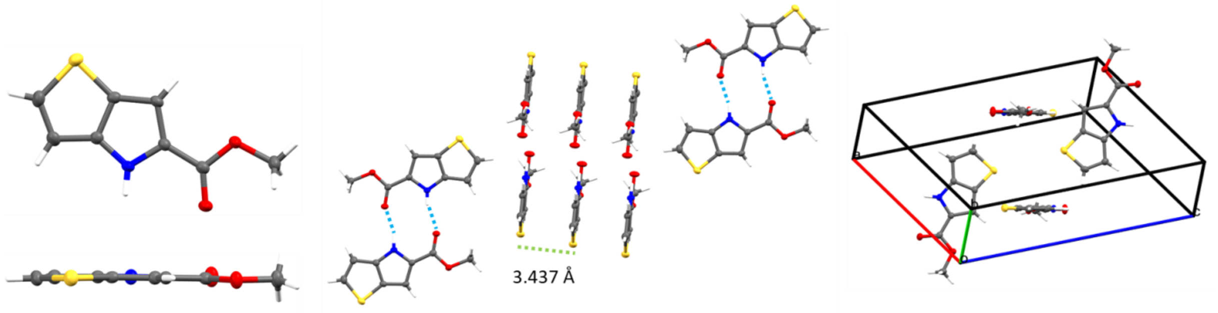

Figure S9. Single crystal X-ray structure analysis of thienopyrrole **25**, front view (top left); side view along the thienopyrrole plane (bottom left); hydrogen bonds between NH- and carbonyl group of adjacent molecules (blue dotted lines) and π–π interactions between the molecules (green dotted line) (middle); unit cell comprising four molecules (right).

Table S1: X-ray structure analysis data of thienopyrrole **25**.

Bond precision:	C-C = 0.0023 A		Wavelength=0.71073
Cell:	a=11.0745(8)	b=4.6972(3)	c=15.6995(9)
	alpha=90	beta=100.785(6)	gamma=90
Temperature:	150 K		

	Calculated	Reported
Volume	802.25(9)	802.26(9)
Space group	P 21/n	P 1 21/n 1
Hall group	-P 2yn	-P 2yn
Moiety formula	C8 H7 N O2 S	C8 H7 N O2 S
Sum formula	C8 H7 N O2 S	C8 H7 N O2 S
Mr	181.21	181.21
Dx,g cm-3	1.500	1.500
Z	4	4
Mu (mm-1)	0.355	0.355
F000	376.0	376.0
F000’	376.66	
h,k,lmax	15,6,21	14,6,21
Nref	2238	1974
Tmin,Tmax	0.915,0.975	0.899,1.000
Tmin’	0.899	

Correction method= # Reported T Limits: Tmin=0.899 Tmax=1.000
AbsCorr = MULTI-SCAN

Data completeness= 0.882 Theta(max)= 29.464

R(reflections)= 0.0367(1601) wR2(reflections)= 0.0855(1974)

S = 1.032 Npar= 111

Table S2: Selected bond lengths of **25**. Atomic labels correspond to Figure S8.

Atom1	Atom2	Bond length/Å
C5	C7	1.357(3)
C7	C4	1.416(2)
C4	C6	1.396(3)
C5	S1	1.739(2)
S1	C6	1.732(2)
C6	C2	1.402(2)
C2	C3	1.385(2)
C3	N3	1.382(2)
N3	C4	1.361(2)
C3	C1	1.448(2)
C1	O1	1.222(2)
C1	O2	1.338(2)
O2	C8	1.445(2)

Table S3: Selected bond angles of **25**. Atom labels correspond to Figure S8.

Atom1	Atom2	Atom3	Angle/deg
S1	C5	C7	114.2(1)
C5	C7	C4	110.0(2)
C7	C4	C6	115.0(2)
C4	C6	S1	109.9(1)
C6	S1	C5	90.79(9)
C4	C6	C2	108.9(2)
C6	C2	C3	105.3(1)
C2	C3	N3	109.9(1)
C3	N3	C4	108.2(1)
N3	C4	C6	107.7(1)
C2	C3	C1	130.7(2)
C3	C1	O2	112.4(1)
C1	O2	C8	115.3(1)
C3	C1	O1	124.7(2)

Table S4: Selected torsion angles of **25**. Atom labels correspond to Figure S8.

Atom1	Atom2	Atom3	Atom4	Torsion angle/deg
N3	C3	C1	O1	2.1(3)
N3	C3	C1	O2	-178.7(1)
C2	C3	C1	O2	1.8(3)
C3	C1	O2	C8	-177.8(1)
S1	C6	C4	N3	-178.9(1)

6. References

1. Sheldrick, G. M. *SADABS* **2002**. Version 2.03. University of Göttingen, Germany.
2. Dolomanov, O.V.; Bourhis, L.J.; Gildea, R.J.; Howard, J.A.K.; Puschmann, H. *J. Appl. Cryst.* **2009**, *42*, 339-341. Sheldrick, G.M. *Acta Cryst.* **2008**, *A64*, 112-122. Sheldrick, G.M. *Acta Cryst.* **2015**, *C71*, 3-8
3. Malatesta, L.; Angoletta, M. *J. Chem. Soc.* **1957**, 1186–1188.
4. Fuller, L. S.; Iddon, B.; Smith, K. A. *J. Chem. Soc. Perkin Trans. 1* **1997**, 3465–3470.
5. Gronowitz, S.; Zhang, Y.; Hörnfeldt, A.-B. *Acta. Chem. Scand.* **1992**, *46*, 654–660.
6. Bélanger, D.; Tong, X.; Soumaré, S.; Dory, Y. L.; Zhao, Y. *Chem. Eur. J.* **2009**, *15*, 4428–4436.
7. Förtsch, S.; Vogt, A.; Bäuerle, P. *J. Phys. Org. Chem.* **2017**, *30*:e3743.
8. Dogru, M.; Handloser, M.; Auras, F.; Kunz, T.; Medina, D.; Hartschuh, A.; Knochel, P.; Bein, T.*Angew. Chem. Int. Ed.* **2013**, *52*, 2920–2924.
9. Zhang, X.; Côté, A. P.; Matzger, A. J. *J. Am. Chem. Soc.* **2005**, *127*, 10502-10503.
10. Brier, E.; Wetzel, C.; Bauer, M.; Mena-Osteritz, E.; Wunderlin, M.; Bäuerle, P. *Chem. Mater.* **2019**, *31*, 7007-7023.
11. Wetzel, C.; Mishra, A.; Mena-Osteritz, E.; Walzer, K.; Pfeiffer, M.; Bäuerle, P. *J. Mater. Chem. C*. **2016**, *4*, 3715-3725.
12. Pankow, R. M.; Munteanu, J. D.; Thompson, B.C. *J. Mater. Chem. C.*, **2018**, 6, 5992-5998.
13. Subramaniyan, S.; Xin, H.; Kim, F. S.; Jenekhe, S. A. *Macromolecules* **2011,** *44*, 6245-6248.
14. O'Brien, A. G.; Lévesque, F.; Seeberger, P. H. *Chem. Commun.* **2011**, *47*, 2688-2690.
15. Lotz, S.; van Jaarsveld, N. A.; Liles, D. C.; Crause, C.; Görls, H.; Terblans, Y. M. *Organometallics* **2012**, *31*, 5371–5383 (ccdc 959540, 798034, 798034).
